# Genetic Cascade Screening for Familial Hypercholesterolemia: A Randomized Clinical Trial

**DOI:** 10.1001/jamanetworkopen.2026.6100

**Published:** 2026-04-13

**Authors:** David Nanchen, Aziz Chaouch, Renzo Scuderi, Vincent Faivre, Thomas von Känel, Georg Ehret, Nathalie Brun, Isabella Sudano, Hans Rickli, Grégoire Girod, Audrey Butty Dettwiler, Kristina Krasieva, Clara Podmore, Nicolas Rodondi, Elisavet Moutzouri, Diana Ballhausen, Rosaria Del Giorno, Arnold von Eckardstein, Jürg H. Beer, Augusto Gallino

**Affiliations:** 1 Center for Primary Care and Public Health (Unisanté), University of Lausanne, Lausanne, Switzerland; 2 Central Institute of the Hospitals, Hôpital du Valais, Sion, Switzerland; 3 Division of Cardiology, Geneva University Hospitals, University of Geneva, Geneva, Switzerland; 4 Children Clinic, Geneva University Hospitals, University of Geneva, Geneva, Switzerland; 5 Department of Cardiology, University Heart Center, Zurich University Hospital, University of Zurich, Zurich, Switzerland; 6 Department of Cardiology, Kantonsspital St Gallen, St Gallen, Switzerland; 7 Department of Cardiology, Hôpital du Valais, Sion, Switzerland; 8 Institute of Family Medicine, Faculty of Science and Medicine, University of Fribourg, Fribourg, Switzerland.; 9 Institute of Primary Health Care (BIHAM), University of Bern, Bern, Switzerland; 10 Department of General Internal Medicine, Inselspital, Bern University Hospital, University of Bern, Bern, Switzerland; 11 Pediatric Metabolic Unit, Woman-Mother-Child Department, Lausanne University Hospital and University of Lausanne, Lausanne, Switzerland; 12 Cardiovascular Research Unit, Dipartimento Medicina Interna, ORBV, Ente Ospedaliero Cantonale, Università della Svizzera Italiana, Switzerland; 13 Institute of Clinical Chemistry, Zurich University Hospital, University of Zurich, Zurich, Switzerland; 14 Department of Molecular Cardiology, Zurich University Hospital, University of Zurich, Zurich, Switzerland

## Abstract

**Importance:**

Familial hypercholesterolemia is a common genetic disorder associated with premature cardiovascular disease. Genetic cascade screening is recommended but remains underused due to privacy laws that prevent clinicians from directly contacting at-risk relatives.

**Objective:**

To examine whether implementing a web-based communication platform in a patient-mediated genetic cascade screening program for familial hypercholesterolemia increases uptake compared with usual care.

**Design, Setting, and Participants:**

This open-label, multicenter, implementation randomized clinical trial (CATCH) was conducted between November 1, 2020, and October 31, 2023, at 7 cardiovascular prevention or lipid clinics across Switzerland, representing French-, German-, and Italian-speaking regions. Adults aged 16 years or older with genetically confirmed familial hypercholesterolemia and at least 1 eligible first-degree relative living in Switzerland participated. A total of 87 index patients with 359 eligible first-degree relatives were randomized by family cluster (1:1) to the intervention or usual care arm.

**Intervention:**

A secure web-based platform allowing participants to send preprepared electronic messages (email or text) to their relatives, linking them directly to participating centers for genetic testing.

**Main Outcomes and Measures:**

The primary outcome was cascade screening uptake, defined as the proportion of eligible first-degree relatives undergoing genetic testing within 6 months. The secondary outcome was new cases identified.

**Results:**

Among 221 adults screened across multiple families, 87 (39.4%) had genetically confirmed familia hypercholesterolemia (median [IQR] age, 49.2 [16.4-83.7] years; 46 [52.9%] female; median [IQR] highest low-density lipoprotein cholesterol, 289.58 [139.00-498.07] mg/dL); 43 were randomized to the usual care arm, and 44 were randomized to the intervention arm. Among the 359 eligible relatives (median family size, 4), 99 (27.6%) underwent genetic testing. Uptake was higher in the intervention arm (30.4%; 95% CI, 22.0%-40.4%) than in usual care arm (16.7%; 95% CI, 10.1%-26.3%), yielding an odds ratio of 2.18 (95% CI, 1.06-4.51; *P* = .03). New case identification was also greater (17.0% vs 8.1%; odds ratio, 2.32; 95% CI, 1.07-5.05; *P* = .03). Newly identified patients were often untreated or had modifiable risk factors.

**Conclusions and Relevance:**

In this randomized clinical trial of patient-mediated genetic cascade screening supported by a web-based platform, participation in genetic testing and detection of familial hypercholesterolemia was increased compared with usual care. These results suggest that the use of digital communication tools enhanced the reach and effectiveness of genetic screening programs within privacy-regulated health systems.

**Trial Registration:**

ClinicalTrials.gov Identifier: NCT04419090

## Key Points

**Question:**

How can the uptake of genetic cascade screening for familial hypercholesterolemia be improved when health care practitioners cannot directly contact relatives?

**Findings:**

In this randomized clinical trial of 87 families with genetically confirmed familial hypercholesterolemia, 30% of relatives in the intervention arm underwent genetic testing when given access to web-based communication technologies compared with 17% in the usual care arm, representing a significant difference.

**Meaning:**

This study’s results suggest that leveraging communication technologies may enhance effectiveness of genetic cascade screening programs for familial hypercholesterolemia.

## Teaser

This randomized clinical trial of families with familial hypercholesterolemia examines whether the addition of a web-based communication platform to usual screening improves participation and detection probabilities among first-degree relatives.

## Introduction

Familial hypercholesterolemia (FH) is a common genetic disorder associated with an increased risk of early-onset myocardial infarctions.^1,2,3^ FH is usually inherited in an autosomal dominant manner, such that first-degree relatives have a 50% probability of carrying the familial pathogenic variant. To promote early detection and treatment, international guidelines consistently recommend genetic cascade screening.^4,5,6,7^ The Centers for Disease Control and Prevention classifies FH as a tier 1 genomic condition, emphasizing its population health relevance.

Despite strong consensus, genetic cascade screening remains inconsistently implemented in routine practice.^8^ Most FH cascade screening programs are local initiatives rather than nationally organized programs, with the exception of some high-income countries.^9^ A major barrier is that privacy regulations restrict health care practitioners from directly contacting at-risk relatives.^10^ When relatives rely solely on information transmitted by affected family members, participation in screening is low and variable.^11,12,13^ Prior uptake estimates originate mainly from noncomparative evaluations of cascade programs, often lacking systematic assessment of the number of eligible relatives per index case, making cross-study comparisons difficult. Additionally, interventions aimed at improving the uptake of genetic cascade screening for FH have not been evaluated in randomized clinical trials.^11,14,15^ Consequently, the most effective patient-mediated strategy compatible with privacy frameworks remains uncertain.^16^ Implementation studies^17,18,19^ are needed before genetic cascade screening can be widely established as standard practice. The CATCH (Cascade Genetic Testing of Familial Hypercholesterolemia in Switzerland) study was a multicenter, open-label, implementation randomized clinical trial^20,21^ that assessed whether adding a web-based communication platform to usual patient-mediated cascade screening could improve participation and detection probabilities among first-degree relatives compared with usual care.

## Methods

### Study Setting

Between November 1, 2020, and January 31, 2023, adults aged 16 years or older with a clinical diagnosis of probable FH, defined by a Dutch Lipid Clinic Network (DLCN) score of 6 points or more, were offered genetic testing.^22,23^ Relatives of index case patients carrying a pathogenic variant were offered testing from January 1, 2021, to October 31, 2023, if 5 years or older. The study took place across 7 cardiovascular prevention or lipid clinics in French-, Italian-, and German-speaking regions of Switzerland. Before this trial, FH genetic testing and cascade screening were rarely practiced due to limited reimbursement. Participating clinicians had expertise in dyslipidemia and cardiovascular prevention and received targeted training on FH inheritance and family counseling. Although patients with FH were not involved in the design, conduct, or reporting of the trial, they contributed to reviewing the content of the preprepared text message and email used for family communication. The study followed the 2025 Consolidated Standards of Reporting Trials (CONSORT) reporting guidelines.^20,21,24,25^ The study was approved by all regional ethics committees. All participants provided written informed consent. The trial protocol can be found in Supplement 1.

### Participants

Index case patients were eligible if they were 16 years or older, had a DLCN score of 6 or greater, and had at least 1 living first-degree relative who resided in Switzerland or was able to attend a study center. A DLCN score of 6 or greater corresponded to a low-density lipoprotein cholesterol (LDL-C) level of 250.97 mg/dL or higher or an LDL-C level of 154.44 mg/dL or higher (to convert to millimoles per liter, multiply by 0.0259) while undergoing treatment with good adherence. Early cardiovascular disease was defined as myocardial infarction, stroke, or revascularized peripheral artery disease before 60 years of age. Exclusion criteria included secondary causes of hypercholesterolemia. Socioeconomic data, lifestyle factors, comorbidities, lipid profiles, physical examination findings, and medication use were recorded using standardized questionnaires and clinical assessments (eMethods 2 in Supplement 2).

Eligible relatives of index case patients with confirmed genetic FH were biological first-degree relatives aged 5 years or older. Relatives living abroad were eligible if the referent mentioned that they would be able to visit a center. Both index case patients and relatives were excluded if they were pregnant, breastfeeding, or unable to follow the study procedures due to language barriers, severe psychiatric disorders, or dementia.

### Cascade Procedures and Family Trees

Index cases with a pathogenic variant received counseling to encourage informing relatives about genetic testing availability (eFigure 1 in Supplement 2). Family trees documenting all first-degree relatives were generated using an electronic case report form standardized across sites (eFigure 2 in Supplement 2). Relatives testing positive also received counseling and had their own family trees completed, ensuring that no individual was counted twice. If one parent tested positive and the other had not yet been tested, the untested parent was not eligible because the origin of the familial variant was already known. Up to 3 cascade cycles were permitted to identify as many affected relatives as possible. Once a relative tested positive for the familial variant, they became a new case patient, and their first-degree relatives were subsequently eligible for genetic testing. Screening stopped for relatives of individuals in whom the familial variant was absent.

### Cluster Randomization

Once a pathogenic variant was identified in an index case patient, the entire family cluster was randomized (block sizes of 4 or 6) to intervention or control stratified by center. Due to the nature of the intervention, blinding of study personnel was not possible. Participants allocated to the control arm did not have access to the web-based platform designed to facilitate family communication.

### Control Arm (Usual Care)

Index case patients and relatives who tested positive were counseled to inform family members through standard patient-mediated communication (eFigure 1 in Supplement 2). They could provide relatives with study contact information and were told that genetic testing was available free of charge.

### Intervention Arm

The intervention combined usual counseling with access to a web-based communication platform (eFigure 3 in Supplement 2). Study personnel were not required to present the platform. After receiving positive genetic results, participants received an email link or a unique code to access the platform. The platform displayed the family members and offered customizable preprepared text and email templates explaining FH and the need for genetic testing (eFigure 4 and eTable 1 in Supplement 2). Each message contained an embedded link that allowed relatives to consent and select a preferred study center. When a relative selected a center, automated notifications were sent to local study personnel, who then arranged testing. Details are reported in eMethods 1 in Supplement 2.

### Genetic Testing

Index case patients underwent next-generation sequencing for pathogenic variants in *LDLR *(OMIM 606945), *APOB *(OMIM 107730), and *PCSK9* (OMIM 607786) using a next-generation sequencing library preparation kit (Devyser) and MiSeq sequencing (Illumina). Variant interpretation followed FH Variant Curation Expert Panel and American College of Medical Genetics and Genomics criteria.^26,27^ Pathogenic or likely pathogenic variants were confirmed via Sanger sequencing and multiplex ligation-dependent probe amplification (MLPA), as appropriate. Variants of uncertain significance were considered negative. Relatives underwent targeted Sanger sequencing or MLPA testing.

### Outcomes

The primary outcome was cascade screening uptake within 6 months, defined as the number of relatives tested divided by the number of eligible relatives. The secondary outcome was detection probability, defined as the number of relatives testing positive divided by the number of eligible relatives. Outcomes were also evaluated at 3 months.

### Sample Size

Based on previous genetic cascade screening programs, assuming 15% participation in the control arm and 30% in the intervention arm, with an intraclass correlation of 2% and mean (SD) family size of 4, 44 families per arm were required for 90% power at 5% significance level.^4,10^ The target sample was 88 index case patients and 350 eligible relatives. The sample size was calculated using a formula for comparing 2 proportions in a cluster-randomized design and validated through generalized estimating equation–based simulations.^28^ No interim analyses were conducted.

### Statistical Analysis

Continuous variables were summarized with medians (IQRs) and categorical variables with numbers (percentages). Comparisons used Kruskal-Wallis and χ^2^ tests, with permutation procedures to account for clustering. Because relatives within a family were not independent, between-arm comparisons used 1000 permutation tests with group assignments permuted at the family level.^29^ When comparing relatives by genetic test result, permutations were instead performed within families.

Participation and detection probabilities with 95% CIs were estimated using generalized estimating equations with a logit link and with family clusters described using an exchangeable correlation structure.^30^ The study arm was included as the only factor in the model. It was not possible to further adjust the model for the center that each referent visited as would be implied by stratified randomization design because, with only few exceptions (3 families), all referents from the same family visited the same center (ie, center did not vary within family). Robust SEs were obtained using the sandwich estimator.^30^ Nonprespecified subgroup analyses examined 3-month outcomes, relative type (parent, sibling, or child), and cascade cycle. Analyses followed the intention-to-treat principle. In addition, a per-protocol analysis, in which relatives in the intervention arm who were contacted outside the platform were reattributed to the control group, was conducted as a sensitivity analysis. All analyses were performed in R, version 4.2.2 (R Foundation for Statistical Computing),^31^ with statistical significance defined as 2-sided *P* < .05.

## Results

### Study Population

**Figure 1.**
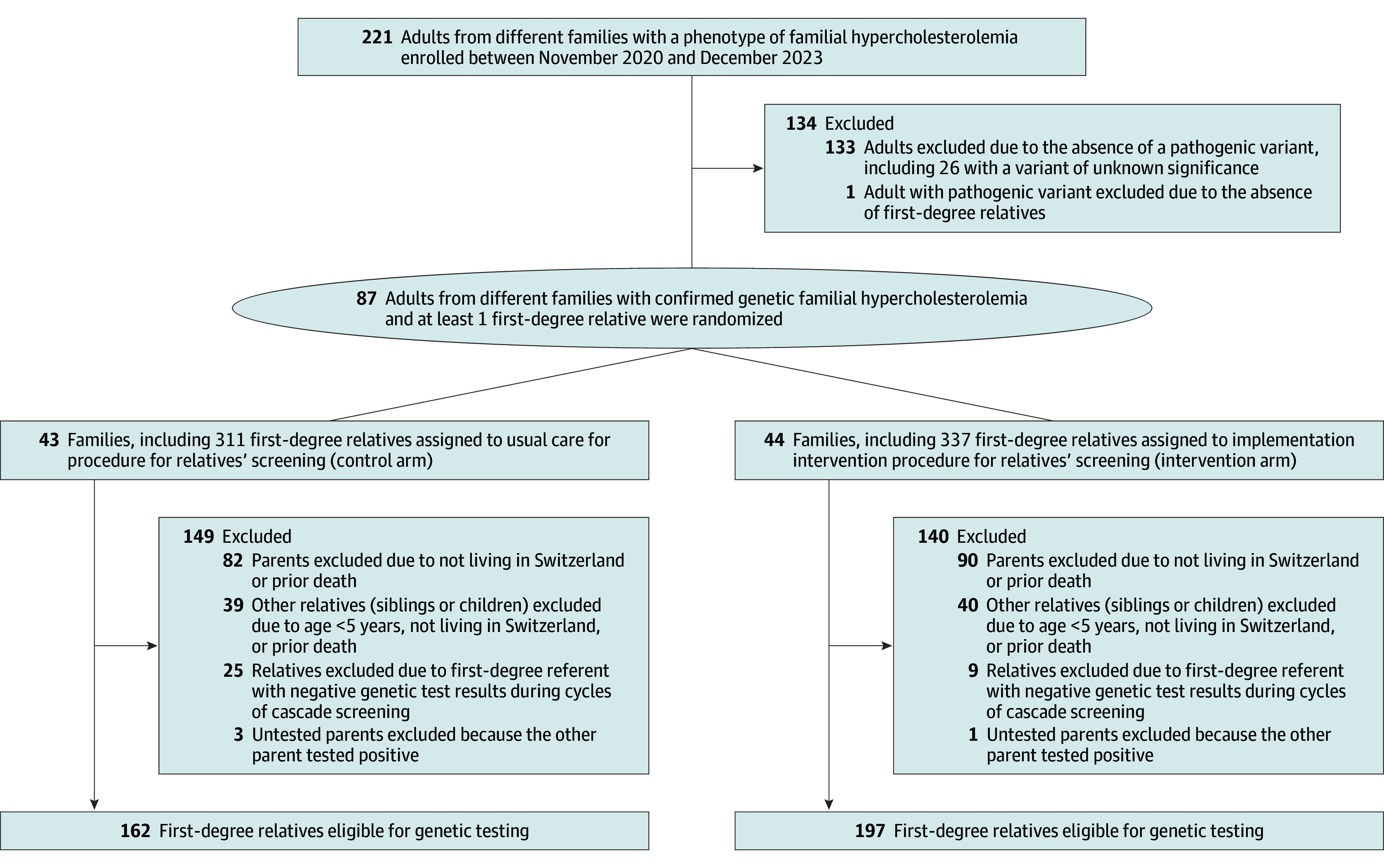
CONSORT Flowchart

Among 221 adults screened across multiple families, 87 (39.4%) had enetically confirmed FH (median [IQR] age, 49.2 [16.4-83.7] years; 46 [52.9%] female and 41 [47.1%] male; median [IQR] highest low-density lipoprotein cholesterol, 289.58 [139.00-498.07] mg/dL). Pathogenic variants were identified predominantly in *LDLR* (65 adults [74.7%]) followed by *APOB* (21 adults [24.1%]) and *PCSK9* (1 adult [1.1%]). One double heterozygote was identified. Of the 87 index case patients, 43 were randomized to the control arm and 44 to the intervention arm (Figure 1). Healthy physical activity was more frequently reported in the intervention arm than in the control arm (38 [88.4%] vs 26 [60.5%]) (Table 1). To account for this imbalance, we conducted a post hoc sensitivity analysis stratified by the level of physical activity of the referent.

**Table 1.** Baseline Characteristics of Index Case Patients With Positive Genetic Test by Study Arm

Characteristic	Index case patients			
	Control arm (n = 43)		Implementation intervention arm (n = 44)	
	Total No.	No. (%)	Total No.	No. (%)
Age, median (IQR), y	43	47.3 (16.4-75.1)	44	52.0 (27.3-83.7)
Sex				
Female	43	25 (58.1)	44	21 (47.7)
Male	43	18 (41.9)	44	23 (52.3)
Socioeconomic status				
High school or university graduation or higher	42	24 (57.1)	42	21 (50.0)
Living alone	42	10 (23.8)	43	6 (14.0)
Married or registered partnership	43	28 (65.1)	44	30 (68.2)
Unemployed or retired	42	11 (26.2)	42	16 (38.1)
Lifestyle				
Smoking status				
Never	41	17 (41.5)	42	26 (61.9)
Former	41	13 (31.7)	42	6 (14.3)
Current	37	11 (26.8)	37	10 (23.8)
Frequent alcohol consumption (≥3 times per week for 6 mo)	37	9 (22.0)	37	12 (28.6)
Higher adherence to Mediterranean diet^a^	43	22 (59.5)	43	23 (62.2)
Healthy physical activity (≥600 MET-min/wk)^b^	42	26 (60.5)	44	38 (88.4)
Comorbidities				
Hypertension^c^	43	10 (23.8)	44	9 (20.5)
Diabetes^d^	43	0	44	4 (9.1)
Cardiovascular diseases^e^	40	9 (20.9)	36	11 (25.0)
Highest recorded lipid parameters, mg/dL				
Total cholesterol	40	359.07 (216.22-555.98)	38	374.52 (212.36-594.59)
LDL-C	38	277.99 (139.00-471.04)	33	297.30 (169.88-498.07)
HDL-C	39	57.92 (30.89-100.39)	34	54.05 (34.75-81.08)
Triglycerides	42	97.35 (53.10-460.20)	43	115.05 (53.10-309.75)
Most recent lipid parameters, mg/dL				
Total cholesterol	42	231.66 (108.11-471.04)	43	235.52 (92.66-621.62)
LDL-C	42	139.00 (42.47-378.38)	43	158.30 (42.47-521.24)
HDL-C	42	57.92 (23.17-115.83)	43	50.19 (27.03-119.69)
Triglycerides	43	88.50 (44.25-433.65)	44	97.35 (35.40-203.55)
Clinical examination				
Xanthomas or xanthelasmas		6 (14.0)		8 (18.2)
Corneal arcus	41	8 (19.5)	43	3 (7.0)
Systolic blood pressure, median (IQR), mm Hg	41	127.0 (96.0-171.0)	43	124.0 (84.0-171.0)
Diastolic blood pressure, median (IQR), mm Hg	41	79.0 (59.0-108.0)	43	76.0 (42.0-97.0)
BMI, median (IQR)	42	24.0 (18.0-34.0)	44	25.0 (15.0-33.0)
Medication				
Lipid-lowering drugs	41	34 (82.9)	43	39 (90.7)
Statins	40	30 (75.0)	42	27 (64.3)
Ezetimibe	40	15 (37.5)	40	20 (50.0)
PCSK9 inhibitors	38	11 (28.9)	39	10 (25.6)
Aspirin	41	8 (19.5)	42	13 (31.0)
Oral anticoagulants	41	8 (19.5)	41	3 (7.3)
Antidepressants	40	2 (5.0)	42	4 (9.5)
Genetics				
Pathogenic variant in the *LDLR* gene	43	31 (72.1)	44	34 (77.3)

Abbreviations: BMI, body mass index (calculated as weight in kilograms divided by the square of height in meters); LDL-C, low-density lipoprotein cholesterol; HDL-C, high-density lipoprotein cholesterol; MET, metabolic equivalent of task.

SI conversion factors: To convert total cholesterol, HDL-C, and LDL-C to millimoles per liter, multiply by 0.0259; triglycerides to millimoles per liter, multiply by 0.0113.

^a^ Defined as adherence to Mediterranean diet above median according to 14-item Mediterranean Diet Adherence Screener.

^b^ Defined according to the International Physical Activity Questionnaire.

^c^ Defined as physician diagnosed, office systolic blood pressure of 140 mm Hg or higher, diastolic blood pressure of 90 mm Hg or higher, or use of blood pressure–lowering drugs.

^d^ Defined as physician diagnosed or use of antihyperglycemic medication or insulin.

^e^ Defined as coronary heart disease, ischemic cerebrovascular disease, or peripheral artery disease.

### Cascade Cycles and Participation

Across 3 cascade cycles, 359 first-degree relatives were eligible, 162 in the control arm and 197 in the intervention arm (Figure 1). Overall, 99 relatives (27.6%) participated: 78 in cycle 1, 20 in cycle 2, and 1 in cycle 3. In 30 families, participation of relatives was limited to a single cascade cycle, and only 8 families progressed beyond 1 cycle. Baseline characteristics of relatives who participated in genetic testing stratified by study arm are presented in eTable 2 in Supplement 2. There was no statistical difference between arms.

### Intention-to-Treat Analysis

Within 6 months, 32 of the 162 eligible relatives in the control arm and 67 of 197 in the intervention arm underwent genetic testing (Table 2). Adjusted for family clustering, participation was 16.7% (95% CI, 10.1%-26.3%) vs 30.4% (95% CI, 22.0%-40.4%), yielding an odds ratio (OR) of 2.18 (95% CI, 1.06-4.51; *P* = .03). Detection probability was likewise higher in the intervention arm (17.0%; 95% CI, 11.4%-24.8%) vs the control arm (8.1%; 95% CI, 4.6%-14.1%) (OR, 2.32; 95% CI, 1.07-5.05; *P* = .03).

**Table 2.** Uptake of Genetic Cascade Screening and Detection of Genetic Familial Hypercholesterolemia by Study Arm

Study arm	No. of index case patients	No. of eligible relatives	No. of tested relatives	Raw participation, %	Estimated participation, % (95% CI)	OR (95% CI)	*P* value
**6-mo participation probability of relatives**							
Overall	87	359	99	27.6	23.7 (17.9-30.6)	2.18 (1.06-4.51)	.03
Control	43	162	32	19.8	16.7 (10.1-26.3)		
Intervention	44	197	67	34.0	30.4 (22.0-40.4)		
**6-mo detection probability of relatives with positive genetic test results**							
Overall	87	359	55	15.3	12.5 (8.9-17.3)	2.32 (1.07-5.05)	.03
Control	43	162	16	9.9	8.1 (4.6-14.1)		
Intervention	44	197	39	19.8	17.0 (11.4-24.8)		

Abbreviation: OR, odds ratio.

### Subgroup Analyses

**Figure 2.**
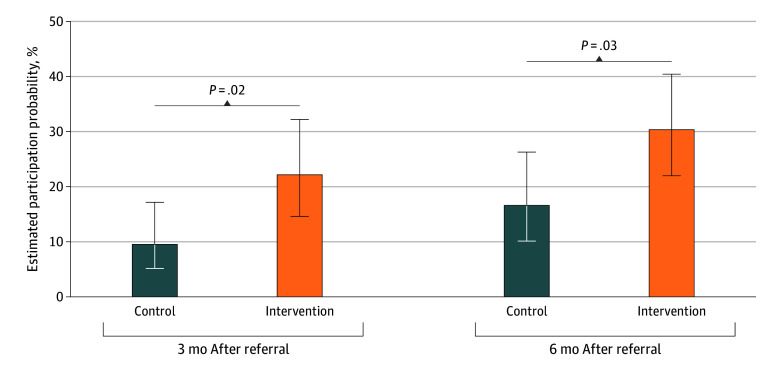
Bar Graph of the Participation Probability of Relatives After 3 and 6 Months by Study Arm Error bars indicate SEs.

**Figure 3.**
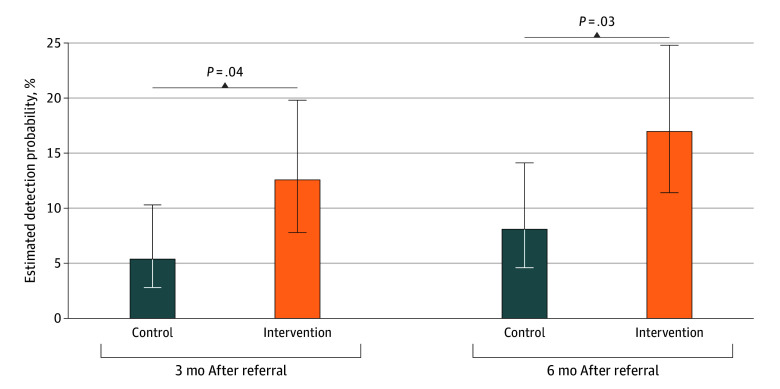
Bar Graph of the Detection Probability of Genetic Familial Hypercholesterolemia After 3 and 6 Months by Study Arm Error bars indicate SEs.

Both arms showed increased participation and detection from 3 to 6 months (Figure 2 and Figure 3). Participation was highest among children compared with siblings and parents (eFigure 5 in Supplement 2). The intervention benefit was most pronounced among siblings and children but not among parents and was limited to the first cascade cycle (eFigure 6 in Supplement 2). The intervention benefit for participation was observed in families in which the referent reported healthy physical activity (OR, 2.70; 95% CI, 1.14-6.37; *P* = .02) but not in those with insufficient activity levels (OR, 0.84; 95% CI, 0.18-3.92; *P* = .83). However, the *P* value for interaction was not statistically significant (*P* = .15) (eTable 3 in Supplement 2).

### Per-Protocol Analysis

In the intervention arm, 21 of 44 index case patients used the web-based platform to contact their relatives. Among 77 contacted relatives, 37 were tested (eTable 4 in Supplement 2). Participation was 18.9% (95% CI, 13.4%-25.9%) in the nonuser group and 43.6% (95% CI, 28.5%-60.0%) in the platform user group (OR, 3.32; 95% CI, 1.53-7.18; *P* = .002). Detection probability similarly improved among platform users, with 10.4% (95% CI, 6.7%-15.6%) in the nonuser group and 21.3% (95% CI, 13.1%-32.7%) in the platform user group (OR, 2.34; 95% CI, 1.11-4.97; *P* = .03).

### Characteristics of Participating Relatives

Among the 99 relatives tested, those with confirmed FH were older (median [IQR] age, 42.0 [5.8-79.4] vs 25.7 [5.2-86.1] years; *P* = .02), had higher levels of LDL-C and triglycerides, and were more likely to have cardiovascular disease than those with a negative genetic test result. Notably, 7 of the 55 FH-positive relatives (12.7%) were younger than 18 years, 14 (28.0%) were current smokers, and 23 (46.0%) were not receiving lipid-lowering therapy (eTable 5 in Supplement 2).

## Discussion

This randomized clinical trial, to our knowledge, provides the first high-quality evidence evaluating the effectiveness of patient-mediated FH genetic cascade screening. The implementation of a web-based communication platform nearly doubled both participation and detection probabilities of FH compared with usual care and proved particularly effective among siblings and children. Importantly, the intervention required no additional health care personnel, respected privacy regulations in settings where health care practitioners are prohibited from directly contacting at-risk relatives, and was scalable across diverse linguistic regions. Participation was associated with actual platform use: in the per-protocol exploratory analysis, relatives contacted through the platform showed substantially higher participation than those contacted by other means. Among newly confirmed FH relatives, smoking prevalence was similar to the general population, and half were not receiving lipid-lowering therapy, underscoring a critical opportunity for early intervention in seemingly healthy individuals at elevated genetic risk.^32,33^

To our knowledge, our study is the only randomized clinical trial to compare 2 strategies for motivating relatives to participate in genetic cascade testing for FH.^16,34^ Most prior studies were noncomparative program evaluations and lacked robust methods to estimate participation.^10,13,18,35^ Notably, participation depends on the number of eligible first-degree relatives per family, which varies by family size and structure. Many studies reported the number of newly identified FH cases per index case, a metric highly influenced by family size and not accounting for clustering.^13^ Others estimated participation as the proportion of contacted relatives who were tested, but contacted relatives are inherently more likely to participate.^10,36^ Only 3 noncomparative FH studies provided data to estimate the number of eligible relatives, yet 2 studies did not use genetic testing and the third lacked detailed information on eligible relatives.^37,38,39^ This heterogeneity limits comparisons across studies.

Accurate estimation of cascade uptake requires knowing the number of eligible relatives through a complete family pedigree. In other genetic conditions, we identified 1 previous multicenter randomized clinical trial from Australia that evaluated the addition of telephone counseling to usual care to improve the uptake of patient-mediated cascade screening.^40^ However, participation was defined as relatives contacting the center rather than undergoing testing, limiting comparability. Nevertheless, in conditions most similar to FH defined as first-degree relatives with elevated transmission risk and with available treatment to reduce genetic risk, the participation probability of relatives was approximately 30%, closely aligning with the highest value we observed in our intervention arm. The overall low levels of participation observed in both our study and prior work highlight an important consideration for effectiveness of universal newborn screening.^41^

Subgroup analyses provided important insights into the cascade process in patient-mediated FH screening. Most families stopped after the first cycle, yet participation was unexpectedly higher in the second cycle, possibly reflecting improved communication once multiple relatives became involved. This suggests that engaging at least 1 relative early may catalyze further participation. Platform benefits were strongest for siblings and children but weaker for reverse cascade attempts toward parents, likely reflecting lower digital engagement among older adults. Only approximately half of index case patients in the intervention arm used the platform to contact relatives, likely reflecting the study’s implementation design, in which study personnel were not required to actively introduce the platform. To improve uptake of cascade screening for FH, active promotion of repeated cascade cycles is needed, supported by an accessible digital communication platform and engagement from health care professionals.

### Limitations

This study has limitations. For privacy reasons, we could not contact or assess the baseline characteristics of relatives who did not participate in genetic testing. Therefore, we were unable to identify all variables associated with participation. Nevertheless, participation was highest among children compared with siblings and parents. Although the intervention required minimal resources, it was conducted under controlled study conditions that may not fully reflect clinical practice. Genetic testing was provided free of charge; however, in many health systems reimbursement remains inconsistent, which may reduce participation outside a trial setting. Therefore, generalizability is greatest in contexts where FH testing is routinely covered. Additionally, blinding of staff was not feasible, leaving possible differences in counseling between arms. Lastly, despite dedicated usability work, the platform could still be improved, particularly for mobile users, as suggested by prior studies.^42,43^

## Conclusions

In this randomized clinical trial of integrating a web-based communication platform into patient-mediated cascade screening, participation in genetic testing and detection of genetically confirmed FH were nearly doubled. Within 6 months, approximately one-third of eligible relatives in the intervention arm were tested, and more than half of them were diagnosed with FH, demonstrating that effective cascade screening was achievable even in settings with strict privacy laws when supported by a secure and user-friendly digital communication tool.

## Notes

Supplement 1.
Trial Protocol

Supplement 2. **eMethods 1. **Web-communication platform **eMethods 2. **Descriptive variables **eTable 1. **Prepared email and text message integrated in the web-communication platform. **eTable 2. **Baseline characteristics of relatives who participated to genetic cascade screening, by study group. **eTable 3. **Uptake of genetic cascade screening of familial hypercholesterolemia stratified by physical activity level of the referent. **eTable 4. **Uptake of genetic cascade screening and detection of genetic familial hypercholesterolemia comparing users and non-users of the web-based communication platform. **eTable 5.** Baseline characteristics of relatives having participated to genetic cascade screening within 6 months of referral, classified by result of genetic test. **eFigure 1. **Procedures and counselling for genetic cascade screening, according to study group. **eFigure 2. **Generating Family Trees from the Participant’s Electronic File **eFigure 3. **Information flow in the implementation intervention group **eFigure 4. **Content of the web-based platform to invite relatives **eFigure 5**. 6-month participation probability of relatives by type of relative and by study arm **eFigure 6**. 6-month participation probability of relatives by screening cycle and by study arm

Supplement 3.
Data Sharing Statement
