# Supplementary material for: Genetic Cascade Screening for Familial Hypercholesterolemia: A Randomized Clinical Trial
Source: JAMA Netw Open. 2026 Apr 13;9(4):e266100. doi: 10.1001/jamanetworkopen.2026.6100 (PMC13077512; doi:10.1001/jamanetworkopen.2026.6100)
Supplement: Supplement 1. — Trial Protocol [file jamanetwopen-e266100-s001.pdf]

Description in protocol V1.3, dated 10.08.2022:

*For the monogenic positive FH group, we calculated that it will be necessary to include **41 families** per group to show a statistically significant difference between the two groups with a significance level of 5% and a power of 90%, assuming that the consent rates for the control and interventional groups will be in mean equal to **0.15** respectively **0.3** and will be at 95% in the intervals **[0.1,0.2] (range 0.05)** respectively **[0.1,0.5] (range 0.2)** and that the number of contactable relatives will be in mean equal to 4.*

All other changes were minor, unrelated to the scientific outcomes, and aimed at strengthening participant data protection in accordance with ethical requirements.

The trial protocol originally included a second randomized controlled trial to be conducted among index cases with negative genetic test results, namely those with probable polygenic familial hypercholesterolemia, using cascade lipid profile testing. However, this additional RCT was not carried out due to insufficient family participation in the pilot study.

Final statistical analysis plan

1. Introduction and purpose

The CATCH study is a prospective randomized open-label multicenter implementation trial that promotes family genetic cascade screening in Switzerland. Each family from an adult older than 16 years with a newly diagnosed pathogenic variant for familial hypercholesterolemia (FH) is randomly assigned to a multigenerational patient-mediated genetic cascade screening (1:1) either via the possibility to use of a web-based platform designed to send pre-prepared text messages or emails, and allowing to connect to a specialized clinical center, or a genetic cascade screening via the local procedure. The aim of the study is to show that the proportion of family members

who participate to the screening among those eligible is higher in the experimental group than in the control group.

2. <u>*Objectives and endpoints*</u>

The primary outcome is the uptake of cascade screening, defined as the number of genetic tests performed by family members within 6 months after referral, over the number of eligible first-degree relatives.

The secondary outcome is the proportion of new cases identified, defined as the number of positive tests detected within 6-months of referral among family members over the number of eligible relatives.

The primary and secondary outcomes must be assessed after accounting for family clusters.

3. <u>*Analysis populations*</u>

The population of interest consists in relatives of positive index cases who live in Switzerland (or are able to visit one of the study centre) and are aged 5 years or older. They must be a first-degree relative of a positive subject in their family, either the index case (cycle 1) or another positive relative (cycle > 1).

The primary and secondary outcomes will be primarily assessed using the Intention-to-treat (ITT) principle. However a per-protocol (PP) analysis will also be carried out for information.

Intention-to-treat (ITT):

All relatives will be assigned to the same group as their index case, irrespective of actual use of the web-based screening platform. In the ITT anaysis, all family members thus belong to the same group.

Per-protocol (PP):

Relatives in the control group will remain in the control group as they have no access to the web-based communication platform. However, those relatives initially assigned to the intervention group but who will be contacted outside of the web-based platform by their referent will also be considered as part of the control group. In the PP analysis, relatives of index cases originally assigned to the intervention group may thus be part of the control or the intervention group, depending on how they were contacted by their referent.

4. Primary analysis

Baseline characteristics of index cases and participating relatives will be summarized using the median and interquartile range for continuous variables, and with counts and percentages for categorical variables. Differences between the intervention and control groups will be assessed using the Kruskal-Wallis test statistic for continuous variables, and the χ^2 test statistic for categorical variables. Characteristics of index cases in the two groups will be compared using standard asymptotic versions of these tests (i.e. for independent data). On the other hand, characteristics of the relatives will be compared after reconstructing the distribution of the test statistics under the null hypothesis using 1000 approximate permutation tests with group allocation permuted at the family level (between-family permutations). When comparing characteristics of relatives depending on the result of the genetic test, permutations of test result will be carried out between relatives from the same family (within-family permutations).

The probabilities of participation and detection as well as their 95% confidence intervals (CI) will be estimated using generalized estimating equations (GEE), using an exchangeable correlation structure to account for the data dependence induced by the family clusters. The model allows estimation of marginal probabilities of participation/detection in each arm (i.e. average probability at the population level), as well as the marginal odds ratio comparing the two arms.

5. Secondary analyses

Subgroup analyses for participation will be performed by stratifying relatives according to the nature of familial relationship with their referent (i.e. parent, sibling or child of the referent), or by the type of referent, to assess the number of cascade cycles that generated the contact (i.e. cycle 1: direct referent is an index case; cycle 2: referent of referent is an index case etc).

6. Handling missing data

No missing data will affect the primary and secondary outcomes. The number of subjects with non-missing values in each demographic variable will be reported in the tables comparing the two groups.

7. Interim analysis

Not applicable

8. Software & output

All statistical analyses will be carried out in R version 4.2.2.

Summary of changes between final and original statistical analysis plan from protocol V1.3, dated 10.08.2022

Sample size:

The sample size calculation originally described in the protocol V1.3, dated 10.08.2022 was slightly revised as follows . The revised calculation assumed an intraclass correlation coefficient of 0.2 to account for family effects. This translates into 95% prediction intervals for family-specific participation probabilities of 9 - 22% in the control arm, and 20 - 41% in the intervention arm. With these adjustment, the required sample size increased to 44 families per arm (vs 41 in the protocol V1.3, dated 10.08.2022).

Modelling:

In the protocol V1.3, dated 10.08.2022, participation to screening (i.e. primary outcome) was modelled using a logistic mixed effect regression model, using the group as fixed effect and the family as random effect. In the final statistical analysis plan, we proposed to rather use generalized estimating equations (GEE) in order to estimate the marginal probability of participation/detection. This change has been motivated for two reasons. First, interest lies primarily in marginal probabilities (i.e. estimated at the population level) rather than conditional probabilities (i.e. within families) as estimated from mixed effect models. Secondly, unlike mixed effects models, GEE are robust to misspecification of the covariance structure in the data, which makes this approach appealing when the covariance structure is not of particular interest.

Reporting

- The per-protocol analysis and the sub-group analyses were not specified in the trial protocol V1.3, dated 10.08.2022. These analyses were therefore newly added in the final statistical analysis plan.

- These three secondary outcomes mentioned in the trial protocol V1.3, dated 10.08.2022 were not reported in the manuscript:
    a. the differences in the use of lipid-lowering drug over 6 months
    b. the change in LDL-cholesterol over 6 months
    c. the transmission rate of phenotype and genotype into families

  These outcomes were not directly associated with the intervention aimed to improve uptake of cascade screening and were therefore not appropriate to include in this randomized controlled trial report. They will be presented as observational outcomes in separate publications.

- Safety endpoint including qualitative research were not collected due to limited resources. As noted in our manuscript on page 6: "As the potential risks of genetic cascade screening for FH have already been examined in qualitative studies and are considered minimal, no specific risks were reported in our trial."

- The trial protocol V1.3, dated 10.08.2022 originally included a second randomized controlled trial to be conducted among index cases with negative genetic test results, namely those with probable polygenic familial hypercholesterolemia, using cascade lipid profile testing. However, this additional RCT was not carried out due to insufficient family participation in the pilot study. This information was reported on page 3 of the supplementary material

Clinical Protocol Template for ClinO, Chapter 4 »Other Clinical Trials«

Cascade genetic testing of familial hypercholesterolemia: the CATCH multicenter randomized controlled trial

---

Study Type:	Other Clinical Trial according to ClinO, Chapter 4
Risk Categorisation:	Risk category A according to ClinO, Art. 61
Study Registration:	1. Registration number from Clincaltrials.gov NCT04419090<br>2. Registration number from the FOPH portal SNCTP (Swiss National Clinical Trial Portal) not yet available<br><br>Unisanté, University of Lausanne<br>Rue du Bugnon 44<br>1011 Lausanne
Principal Investigator	Pr Dr med David Nanchen<br>Unisanté, University of Lausanne<br>Rue du Bugnon 44<br>1011 Lausanne
Investigated Intervention:	Cascade screening of family members of an index case with familial hypercholesterolemia
Protocol ID	NCT04419090 (Clinicaltrial.org)
Version and Date:	Version 1.3 (dated 10/08/2022)

CONFIDENTIALITY STATEMENT

If applicable, add an institutional confidentiality statement here respecting that it is not in conflict with the applicable transparency rules.

e.g. “The information contained in this document is confidential and the property of Dr David Nanchen (or “the Principal Investigator”). The information may not - in full or in part - be transmitted, reproduced, published, or disclosed to others than the applicable Competent Ethics Committee(s) and Regulatory Authority(ies) without prior written authorisation from the Principal Investigator except to the extent necessary to obtain informed consent from those who will participate in the study.

PROTOCOL SIGNATURE FORM

Study Title	Cascade genetic testing of familial hypercholesterolemia: the CATCH randomized controlled trial
Study ID	CATCH study

The SponsorInvestigator has approved the protocol version 1.2 (dated 03/08/2020) and confirm hereby to conduct the study according to the protocol, current version of the World Medical Association Declaration of Helsinki, and ICH-GCP guidelines as well as the local legally applicable requirements.

Sponsor and Principal Investigator:

Name: *Pr Dr med David Nanchen,*
Centre universitaire de médecine générale et santé publique (Unisanté)
Université de Lausanne
Rue du Bugnon 44
1011 Lausanne

Date: __________________________ Signature:

Local Principal Investigator at study site:

1) Site: Cardiovascular prevention and cholesterol clinic, Center for Primary Care and Community Medicine (Unisanté), University of Lausanne, Lausanne

Principal Investigator: *David Nanchen*

Date: ____________________________ Signature: ______________________________

2) Site: Lipid and cardiovascular clinic, Department of Medicine, *HUG Geneva*

Principal Investigator: *Georg Ehret*

Date: ____________________________ Signature: ______________________________

3) Site: Children Lipid clinic, Pediatric cardiology, *HUG Geneva*

Principal Investigator: *Nathalie Brun*

Date: ____________________________ Signature: ______________________________

4) Site: Lipid clinic, Department of General Internal Medicine, University Hospital of Bern

Principal Investigator: *Nicolas Rodondi*

Date: ____________________________ Signature: ______________________________

5) Site: Prevention clinic, Department of cardiology, University of Zurich

Principal Investigator: *Isabella Sudano*

Date: ____________________________ Signature: ______________________________

6) Site: Centre hospitalier du Valais romand, cardiologie, Sion

Principal Investigator: *Grégoire Girod*

Date: ____________________________ Signature: ______________________________

7) Site: Ospedale San Giovanni, Bellinzona

Principal Investigator: Augusto Gallino

Date: ____________________________ Signature: ______________________________

8) Site:, Cardiology clinic, Cantonal Hospital of St.Gallen*, St-Gallen*

Principal Investigator: *Hans Rickli*

Date: ____________________________ Signature: ______________________________

9) Site:, Service of Pediatrie, CHUV*, Lausanne*

Principal Investigator: *Diana Ballhausen*

Date: ____________________________ Signature: ______________________________

TABLE OF CONTENTS

TABLE OF CONTENTS 3

GLOSSARY OF ABBREVATIONS 5
1 STUDY SYNOPSIS 6
2 BACKGROUND AND RATIONALE 9
3 STUDY OBJECTIVES AND DESIGN 10
3.1 Hypothesis and primary objective 10
3.2 Primary and secondary endpoints 10
3.3 Study design 10
3.4. Study intervention 12
4 STUDY POPULATION AND STUDY PROCEDURES 12
4.1 Inclusion and exclusion criteria, justification of study population 14
4.2 Recruitment, screening and informed consent procedure 15
4.3 Study procedures 16
4.4 Withdrawal and discontinuation 22
5 STATISTICS AND METHODOLOGY 23
5.1. Statistical analysis plan and sample size calculation 23
5.2. Handling of missing data and drop-outs 23
6 REGULATORY ASPECTS AND SAFETY 24
6.1 Local regulations / Declaration of Helsinki 24
6.2 (Serious) Adverse Events 24
6.3 (Periodic) safety reporting 25
6.4 Radiation 25
6.5 Pregnancy (if applicable) 25
6.6 Amendments 25
6.7 (Premature) termination of study 26
6.8 Insurance 26
7 FURTHER ASPECTS 26
7.1 Overall ethical considerations 26
7.2 Risk-benefit assessment 27
8 QUALITY CONTROL AND DATA PROTECTION 27
8.1 Quality measures 27
8.2 Data recording and source data 27
8.3 Confidentiality and coding 28
8.4 Retention and destruction of study data and biological material 28
9 MONITORIMG AND REGISTRATION 29
10. FUNDING / PUBLICATION / DECLARATION OF INTEREST 29
10 REFERENCES 30
Appendix 1: Schedule of assessments (if applicable) 32

GLOSSARY OF ABBREVATIONS

AE	*Adverse Event*
ASR/DSUR	*Annual Safety Repot / Development Safety Report*
BASEC	*Business Administration System for Ethical Committees*
CRF	*Case Report Form*
CTCAE	*Common Terminology Criteria for Adverse Events*
FADP	*Federal Act on Data Protection (in German: DSG, in French: LPD, in Italian: LPD)*
eCRF	*electronic Case Report Form*
FOPH	*Federal Office of Public Health*
GCP	*Good Clinical Practice*
HRA	*Human Research Act (in German: HFG, in French: LRH, in Italian: LRUm)*
ICH	*International Conference on Harmonisation*
ClinO	*Ordinance on Clinical Trials in Human Research (in German: KlinV, in French: OClin, in Italian: OSRUm)*
SAE	*Serious Adverse Event*
FH	*Familial Hypercholesterolemia*

Please expand the list of abbreviations as needed.

1 STUDY SYNOPSIS

**Sponsor / Sponsor-Investigator**	Pr Dr med David Nanchen<br>Unisanté, University of Lausanne<br>Rue du Bugnon 44<br>1011 Lausanne
**Study Title**	Cascade genetic testing of familial hypercholesterolemia: the CATCH randomized controlled trial
**Short Title / Study ID**	CATCH study
**Protocol Version and Date**	Version 1.3 (dated 10/08/2022)
**Study Registration**	Registration number from Clincaltrials.gov not yet available
**Study Category and Rationale**	Other Clinical Trial and Risk category A according to ClinO
**Background and Rationale**	Familial hypercholesterolemia (FH) is a genetic disorder associated with an increased risk of early-onset myocardial infarctions. In Switzerland there are about 40,000 subjects with FH, but only a minority of them are known. Unfortunately, many patients with FH are only diagnosed at the time of hospitalization for acute coronary syndrome. Because effective lipid-lowering drug can reduce the cardiovascular risk, this underdiagnosis is a major missed opportunity for prevention. To improve detection of patient with FH, the use of genetic test has been proposed. Indeed, because the monogenic mutation for FH is autosomal dominant, a cascade screening program to identify at-risk relatives should be therefore very effective. However, the implementation of a multicenter genetic cascade screening program for FH has never been tested in Switzerland.
**Risk / Benefit Assessment**	The rational to support genetic testing for FH are: 1) individuals with monogenic *positive* FH have an increased risk of cardiovascular disease, 2) the availability and efficacy treatments to lower LDL-c levels and cardiovascular risk, and 3) a potential improved medication compliance in the presence of a genetic diagnosis, and 4) increase performance of cascade screening among relatives. The risk of the intervention is related to the psychological effects of unsolicited contact by the cascade screening program. This include a breach in the potential “right not to know” leading to anxiety. The stigmatization of genetic information to obtain a life insurance may also be an issue.
**Objective(s)**	To test in a multicenter open-label randomized controlled trial across Switzerland whether a cascade screening programme for FH, in comparison with usual care, will increase the detection rate of FH within families.
**Endpoint(s)**	The primary outcome will be the difference in the yield of detection of familial hypercholesterolemia (FH) between arms. The yield of detection is the number of test performed/number of contactable relatives.<br>Secondary endpoints include the transmission rate of phenotype and genotype into families. After 6 months follow-up, the differences in the use of lipid-lowering drug and change in LDL-cholesterol will be the third endpoint.
**Study Design**	Multicenter open-label randomized controlled trial, with 2x 2 arms. Index cases with clinical criteria for FH will be separated into two groups before randomization, based on the result of the genetic test, either positive or negative for a mongenic mutation in one of the three genes causing FH. In each of these two groups, a randomization procedure will allocate index cases and their family member into the intervention arm or the control arm to evaluate the best method to contact at-risk relatives and perform cascade screening. In the intervention arm, the index case will be actively supported by a web-based centralized service to initiate contact with their first-degree relatives. In the control arm or “usual care arm”, the index case will be encouraged to initiated contact with their first-degree relatives, but without additional support. The presence or absence of a monogenic mutation in the index case will determine the screening test used for cascade screening of relatives, either genetic or lipid test.
**Statistical Considerations**	The sample size has been calculated according to the primary outcome, for each of the study groups, monogenic *positive* and *negative* FH. For the monogenic *positive/negative* FH group, we calculated that it will be necessary to include 41/36 families per arm to show a statistically significant difference of 20% in the consent rate between the control and interventional arm.<br>These sample sizes were calculated analytically based on the standard errors of the mean proportions and were verified using simulations by the statistician team of Unisanté.

**Inclusion- / Exclusion Criteria**	Inclusion criteria: patients with severe hypercholesterolemia and familial or personal history of early-onset cardiovascular disease = Dutch Lipid Clinic Network score (DLNC) >= 6 points.<br>Exclusion criteria: patients without at least one contactable first-degree family members
**Number of Participants with Rationale**	We will include 82 index cases with monogenic *positive* FH, as well as 72 index cases with monogenic *negative* FH, for a total of 154 index cases. In the intervention arms, we estimate that four relatives per index case will participate to the screening programme. In the control arms, we estimated that two relative per index case will contact the study center for cascade screening. Thus we plan to include a total of about 400 participants in the study, including relatives.
**Study Intervention**	The intervention arm will consist in three 3 cycles of cascade screening of family members. The contact of relatives will be initiated by the index case and supported by a physician and a web-based centralized service. The index case will be provided with a prepared email or SMS message to be further addressed to his first-degree relatives. The email/message will contain a link to a secured web application with a code for the connection. The app will provide information about the transmission mode of FH, the cardiovascular risk associated with FH and the way how to reduce this risk. The relative can then fill out information -first name, last name, Email, phone number- and provide agreement to be contacted for the study. The nearest specialized clinic will then contact the relative to organize further screening.
**Control Intervention**	In the control arm or “usual care arm”, the index case will also be counselled to encourage FH screening among relatives and he will be provided with information about FH, and how to contact information the study center. However, there will be no prepared email or SMS message to be sent to relatives and no support of the centralized service.
**Study procedures**	All index cases included in the study will have a genetic test to diagnose FH. Genetic tests will be performed in the certified central lab of Instituts des Hopitaux Valaisans in Sion, and will be performed to identify a monogenic mutation in three FH genes, namely LDLR, APOB and PCSK9. No other genetic abnormalities will be assessed, but a genetic biobank will be created in Sion for further research projects. If a monogenic mutation is identified, cascade screening of relatives will be done with lipid and genetic tests. If a monogenic mutation is not identified, cascade screening will be done with lipid tests only. The identification and the inclusion of the index case, as well as the pre and post visit counselling for genetic test will be performed by specialized lipid or cardiology centers in Switzerland. Clinicians involved in these study centers will be trained on how to inform patients about the consequences of FH genetic tests for their families.
**Study Duration and Schedule**	Inclusion of index cases: 30 months<br>Study duration for an index case: 8 months<br>Planned 09/2020 of First-Participant-In<br>Planned 12/2024 of Last-Participant-Out, including relatives
**Investigator(s)**	1) Prof Dr. med David Nanchen, Center for Primary Care and Public Health, University of Lausanne<br>2) Prof. Dr. med Augusto Gallino, Ospedale San Giovanni, 6500 Bellinzona<br>3) Prof. Dr. med Beer Jürg Hans, Cantonal Hospital of Baden
**Study Center(s)**	Main Center and centralilzed service: Center for Primary Care and Public Health (Unisanté), University of Lausanne<br>Number of centers to be involved in Switzerland : 10
**Data privacy**	To create family trees and identify parental links of index cases and relatives from different study sites across Switzerland, we will use specific databases elaborated by the informatic team of Unisanté (Julien Thabard). We will attribute to each participant two random participation codes: the PID and the SID code to guarantee privacy of data.
**Ethical consideration**	FH monogenic mutations are transmitted with an autosomal dominant mode. Therefore, each first degree relative of an index case has a 50% chance of having the disorder. Because FH increase the life-long cardiovascular risk and can be effectively treated with lipid-lowering drugs and appropriate diet, physicians should ideally inform relatives of an index case of their potential cardiovascular risk and their options to reduce it. However, legal protection to guarantee privacy of data do not authorize physicians to directly contact at-risk relatives of an index case. Alternatively, the index case can be counselled to inform his relatives about the risk of FH. However this process can be hampered by inefficient family communication. Therefore, ethical tension exists between motivation to promote health care and interest in maintaining privacy of health information. Based on previous studies and on the current

	practice in Switzerland, we have designed a cascade screening program ethically acceptable. First, the procedure of contact will respect autonomy and privacy of at-risk relatives because it will be initiated only by the index case. Second, the study team will contact a relative only when information about willingness to be contacted has been obtained directly by the relative, and not by the index case. Furthermore, qualitative assessment will be performed in a subgroup of relatives in order to identify psychological risks of unsolicited contact, such as the potential for "right not to know", anxiety or stigmatization. Finally, the control arm will be provided with counselling and information to contact relatives, according to clinical guidelines. Children from age of 5 years old could be included as relative of an index case based on current medical practice and clinical guidelines. Indeed, diet recommandations should be initiated at a early age to be effective to reduce the life-long cardiovascular risk in children with FH.
**GCP Statement**	This study will be conducted in compliance with the protocol, the current version of the Declaration of Helsinki, the ICH-GCP, the HRA as well as other locally relevant legal and regulatory requirements.

2 BACKGROUND AND RATIONALE

Familial hypercholesterolemia (FH) is a genetic disorder associated with an increased risk of early-onset myocardial infarctions.[1] In Switzerland there are about 40,000 subjects with FH, but only a minority of them are known.[2,3] Unfortunately, many patients with FH are only diagnosed at the time of hospitalization for acute coronary syndrome. Because effective lipid-lowering drug can reduce the cardiovascular risk, this underdiagnosis is a major missed opportunity for prevention.[4]

FH is transmitted to family members with a autosomal dominant mode. Therefore, each first degree relative of an index case has a 50% chance of having the disorder. To improve detection of patient with FH, the use of genetic test and cascade screening programs among relatives has been recommended by recent European and US guidelines for dyslipidemia and FH.[5,6] The Centers for Disease Control also classified FH as a Tier 1 condition, and recommend the implementation of cascade screening as outlined in the National Institute for Health and Clinical Excellence (NICE) guidelines for identification and management of FH.[7]

However, little is known about effectiveness of a FH screening programme in Switzerland. The implementation of a multicenter cascade screening program for FH has never been tested. First, it is unknown what would be the best method to contact at-risk relatives to improve the yield of FH detection.[8] Because FH increase the life-long cardiovascular risk and can be effectively treated with lipid-lowering drugs and appropriate diet, physicians should ideally inform relatives of an index case of their potential cardiovascular risk and their options to reduce it. However, legal protection to guarantee privacy of data do not authorize physicans to directly contact at-risk relatives of an index case. Alternatively, the index case can be counselled to inform his relatives about the risk of FH. However this process can be hampered by inefficient family communication. Therefore, ethical tension exists between motivation to promote health care and interest in maintaining privacy of health information. This study is designed to test a contact program of relatives initiated by the index case, but actively supported by the healthcare team.

Second, only a proportion of patient with clinical criteria for FH had a known genetic mutation in the LDLR, APOB or PCSK9 gene (monogenic *positive* FH). The yield of FH detection of a cascade screening programme for patient with monogenic *negative* FH remains largely unknown. This study is design to study family cascade screening of all patients with a phenotype of FH, independently of the identification of a currently known monogenic mutation.

This study is risk category A according to ClinO, Art. 61, because the health intervention studied is a cascade screening programme for FH. Genetic cascade screening programme has been associated with only minimal psychological risks and burdens,[8,9] and is supported by clinical guidelines in the US and in Europe.[6,7] The rational to support genetic cascade screening for FH are: 1) individuals with monogenic *positive* FH have an increased risk of cardiovascular disease compared to monogenic *negative* FH patient, 2) the availability and efficacy treatments to lower LDL-c levels and cardiovascular risk, and 3) a potential improved medication compliance in the presence of a genetic diagnosis.[5] 4) identification of family members with monogenic mutations with only modestly elevated LDL-C who would remain undiagnosed if only LDL-C levels are obtained. Therefore, the study will be instrumental in enhancing awareness about necessity to introduce FH genetic screening and cascade screening in Switzerland.

3 STUDY OBJECTIVES AND DESIGN

3.1 Hypothesis and primary objective

We hypothesized that a FH cascade screening program initiated by the index case and supported by a physician and a centralized service will improve the detection rate of FH, compared to usual care.

The aim is to test in a multicenter open-label randomized controlled trial across Switzerland whether a cascade screening programme for FH, in comparison with usual care, will increase the detection rate of FH within families.

3.2 Primary and secondary endpoints

The primary outcome will be the difference in the yield of detection of familial hypercholesterolemia (FH) between arms. The yield of detection is the number of test performed/number of contactable relatives, and will be assessed 6 months after the counselling visit for screening of relatives. Contactable relatives are defined as first-degree family members living in Switzerland.

Secondary endpoints include
- The differences in the number of FH patients identified in each study group. FH patients will be defined according to the Dutch Lipid Network Score of 3 or above (possible, probable or definite FH).[6]
- the differences in the use of lipid-lowering drug over 6 months
- the change in LDL-cholesterol over 6 months
- the transmission rate of phenotype and genotype into families

Safety endpoint include a qualitative assessment of patients' perception and experiences of FH cascade screening.

3.3 Study design

Multicenter open-label randomized controlled trial, with 2x 2 arms (See **Figure 1**). Index cases with clinical criteria for FH will be separated into two groups before randomization, based on the result of the genetic test, either *positive* or *negative* for a mongenic mutation in one of the three genes causing FH: LDLR, APOB, or PCSK9. Thus there will be two groups before randomization for the intervention:
1) the monogenic *positive* FH group, and
2) the monogenic *negative* FH group.

In each two groups, a randomization procedure will allocate index cases and their family member into the intervention arm or the control arm to evaluate the best method to contact at-risk relatives and perform cascade screening. The presence or absence of a monogenic mutation in the index case will determine the screening test used for cascade screening of relatives, either genetic or lipid test or both.

Figure 1

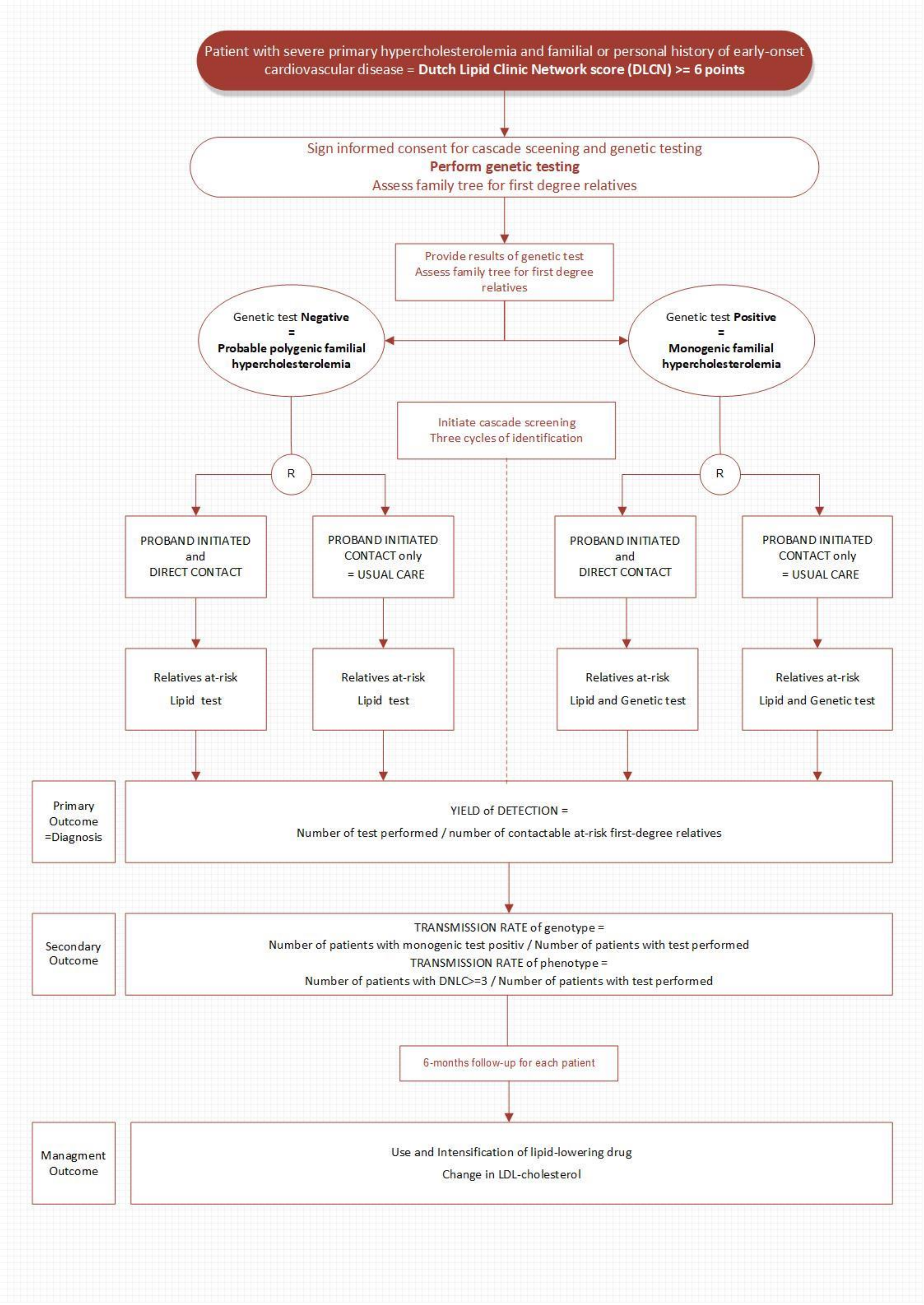
Patient with severe primary hypercholesterolemia and familial or personal history of early-onset cardiovascular disease = Dutch Lipid Clinic Network score (DLCN) >= 6 points
Sign informed consent for cascade sceening and genetic testing
Perform genetic testing
Assess family tree for first degree relatives
Provide results of genetic test
Assess family tree for first degree relatives
Genetic test Negative = Probable polygenic familial hypercholesterolemia
Genetic test Positive = Monogenic familial hypercholesterolemia
Initiate cascade screening
Three cycles of identification
R
R
PROBAND INITIATED and DIRECT CONTACT
PROBAND INITIATED CONTACT only = USUAL CARE
PROBAND INITIATED and DIRECT CONTACT
PROBAND INITIATED CONTACT only = USUAL CARE
Relatives at-risk
Lipid test
Relatives at-risk
Lipid test
Relatives at-risk
Lipid and Genetic test
Relatives at-risk
Lipid and Genetic test
Primary Outcome =Diagnosis
YIELD of DETECTION =
Number of test performed / number of contactable at-risk first-degree relatives
Secondary Outcome
TRANSMISSION RATE of genotype =
Number of patients with monogenic test positiv / Number of patients with test performed
TRANSMISSION RATE of phenotype =
Number of patients with DNLC>=3 / Number of patients with test performed
6-months follow-up for each patient
Managment Outcome
Use and Intensification of lipid-lowering drug
Change in LDL-cholesterol

3.4. Study intervention

The intervention is designed to perform three cycles of cascade screening through several generation of family members of an index case (see **Figure 2**). The procedure for contacting at-risk relatives and perform cascade screening will be similar for monogenic *positive* or *negative* FH.

Figure 2: Example of cascade screening for FH using family tree[10]

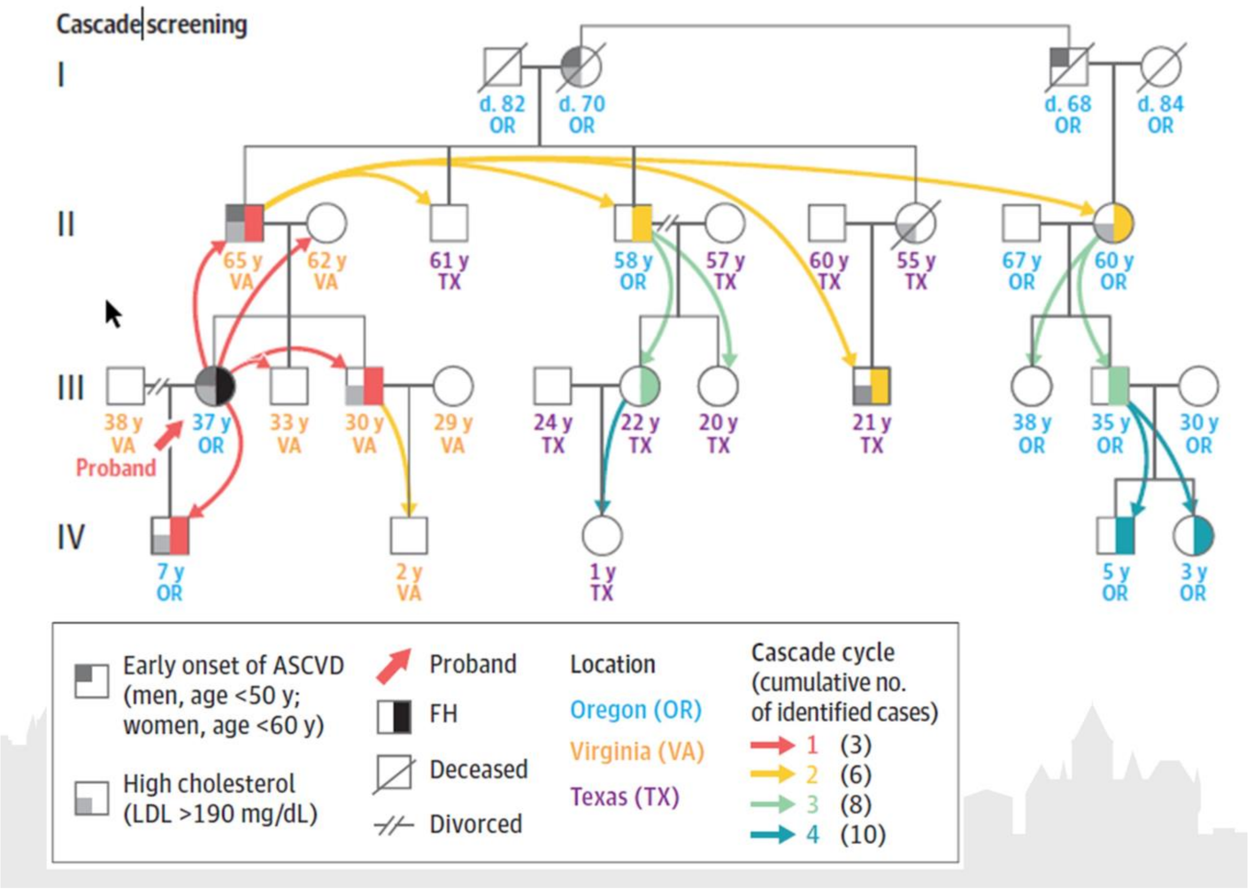

In the intervention arm, the contact of relatives will be initiated by the index case and supported by a web-based centralized service. The centralized service will be hosted by Unisanté in the IT infrastructure of the DSI-CHUV in Lausanne. The index case will be provided with a prepared email or SMS message that the index case can further forward to his first-degree relatives. The email/message will contain a link to a secured web application with a code for the connection (**Figure 3**). By clicking on the link, the relative will connect to a specifically designed CATCH family tree app. The app will provide information about the transmission mode of FH, the cardiovascular risk associated with FH and the way how to reduce this risk. The relative can then fill out information -first name, last name, Email, phone number- and provide agreement to be contacted for the study by one of the study center. The chosen study center will then contact the relative to sign the study informed consent form and organize further screening with similar processes. As described in chapter 8.3, personal identifiable data will be registered in the dedicated REDCap database only after participants signed the study informed consent form.

In the control arm or “usual care arm”, the index case will also be counselled to encourage FH screening among relatives and he will be provided with information about FH and how tocontact the local study center, as recommended by guidelines.[5,6] However, there will be no prepared email or SMS message to be forwarded to relatives and no support of the centralized service from Lausanne.

Several contact procedures have been evaluated by qualitative research[8,9] and have been shown to be without major adverse effect and ethically acceptable.[11,12] Based on these studies and based on the current practice in Switzerland, we have designed a cascade screening program we consider ethically and legally acceptable for four reasons:

1) The procedure of contact will respect the autonomy and privacy of at-risk relatives. The contact will be initiated by the index case, who will be given an adequate opportunity to communicate with family members.
2) The study team will contact a relative only when information about willingness to be contacted has been obtained directly by the relative, and not by the index case.
3) The control arm will also receive information about FH cascade screening as recommended by latest dyslipidemia guidelines.[5,6]
4) Qualitative assessment will be performed in a subgroup of relatives in order to identify psychological risks of unsolicited contact, such as the potential for “right not to know”, anxiety or stigmatization.

Figure 3 : procedure to contact relatives using the web-based centralized service in the intervention arm

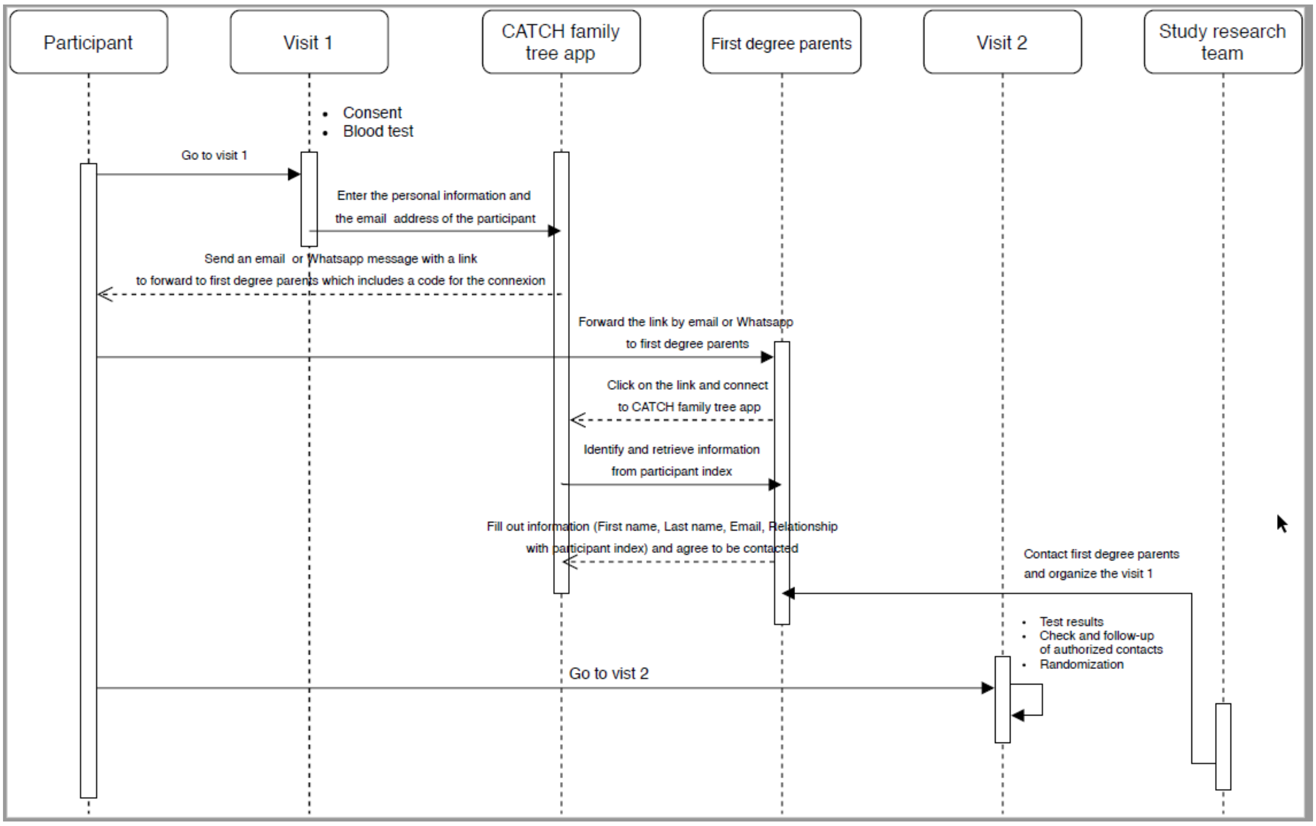

Alternative procedure in the intervention arm, in case of absence of use of email or SMS:

1) After announcing the results of the genetic test to the index case, the physician will provide invitation letters to be send directly to first-degree relatives. The index case will fill the name and address of first degree relatives, sign the letter, and post them free of charge.
2) The invitation letter will contain an information sheet and a paid response coupon. The information sheet will explain the transmission mode of FH, the cardiovascular risk associated with FH, the way how to reduce this risk, as well as the procedure how to organize further screening.
3) The invitation letter will contain the contact information of the study centers and of the centralized service. Response will be possible by pre-paid letter to the centralized service, by e-mail, by SMS or by telephone to the centralized service in Lausanne or to the local study centers.
4) If contacted, the centralized service in Lausanne will help a relative to find a local study center in Switzerland to organize FH screening.

4 STUDY POPULATION AND STUDY PROCEDURES

4.1 Inclusion and exclusion criteria, justification of study population

Inclusion criteria of the index case are based on existing clinical criteria for FH from the Dutch Lipid Clinic Network (DNLC) score, as recommended by lipid guidelines.[6] Only adults patients with severe hypercholesterolemia and familial or personal history of early-onset cardiovascular disease = Dutch Lipid Clinic Network score (DLNC) >= 6 points will be included (see **Appendix 2**). This cutoff correspond to the current international recommendation to perform a genetic test for FH.[6] In most of the case, this correspond to a documented LDL-c of 6.5 mmol/l or above without treatment at least at one occasion. We assume a 40% reduction in LDL-c with lipid-lowering therapy. Thus, under treatment with good adherence, the LDL-c for inclusion will be 4.0 mmol/l or above. Early-onset cardiovascular disease refer to a myocardial infarction, stroke or revascularized peripheral artery disease below 60 years old for men or women.

As relatives, we will also include children from 5 years old to 15 years old, as recommended by clinical guidelines for children with FH.[13] The rationale to identify children with FH is that lifestyle intervention is particularly effective if started early in life. In specific situation, a lipid-lowering treatment can also be initiated from 10 years old.[13] As the DLNC score is not valid for children, we will use the recommended cutoff of LDL-cholesterol to identify children with FH, that is a documented LDL-cholesterol of 4 mmol/l or above without lipid-lowering treatment, or a LDL of 3.5 mmol/l in case of a family history of LDL-c above 6.5 mmol/l among one of the parents. For children, we assume a 30% reduction in LDL-c with lipid-lowering therapy. Thus, under treatment, the LDL-c for inclusion will be 3.0 mmol/l or above.

Inclusion-exclusion criteria for the index case

Inclusion criteria of the index case are

- Adults >=16 years old
- a DLNC >= 6 points or a documented untreated/treated LDL-c of 6.5/4.0 mmol/l or above (see paragraph above for details).
- Ability to give informed consent as documented by signature

Exclusion criteria of the index case include:

- Patients without at least one contactable first-degree family members. Contactable family members include those living in Switzerland. Family members living outside Switzerland will not be considered, except if they can have access to a genetic test for FH.

- Patients with a known medical condition other than FH that contribute to hyperlipidemia (untreated hypothyroidism, nephrotic syndrome, cholestasis, hypopituitarism)
- Pregnant or lactating women
- Inability to follow the procedures of the study, e.g. due to language problems, psychological disorders, dementia, and not having an email address.

Inclusion-exclusion criteria for relatives of an index case

The study will recruit adults and children age >= 5 years in the cascade screening programme, who are relatives of the index case.

Inclusion criteria of the relatives include:

- Age >=5 years old
- To be a first-degree relative of an index case included in the study
- Ability to give informed consent as documented by signature; children younger than 16 years will be enrolled only with the explicit consent of a parent or legal guardian

Exclusion criteria of the relatives include:

- Living outside Switzerland, except if they are leaving close to the border and a medical visit to one of the study center would be feasible
- Pregnant or lactating women, because cholesterol values are not interpretable during pregnancy.
- Inability to follow the procedures of the study, e.g. due to language problems, psychological disorders, dementia. (having an email address is not mandatory for relatives)

We will include 82 index cases with monogenic *positive* FH, as well as 72 index cases with monogenic *negative* FH, for a total of 154 index case. Furthermore, we assume that about 250 patients with clinical criteria of FH will need to be screened to identify 82 index cases with monogenic *positive* FH. Out of the about 200 patients with monogenic *negative* FH, the first 72 will be included. Therefore, about 130 patients with monogenic *negative* FH will not be proposed for cascade screening within this study once the sample size has been achieved.

In the two intervention groups, we estimate that four relatives per index case will participate to the screening programme. In the two control groups, we estimated that two relative per index case will contact the study center for cascade screening. Thus we plan to include a total of about 400 participants in the study, including relatives.

4.2 Recruitment, screening and informed consent procedure

There will be 2 possibilities for screening to ensure successful identification of FH index cases

1) study center screening and 2) laboratory record screening

1) *Study center record screening*: Clinic records from study centers will be examined to identify potential eligible participants with FH. If no prospective visit has been planned at the study center, those patients will be contacted by letter or email to explain the possibility for FH genetic screening within the study protocol. The letter or email will contain the information and consent forms of the study.
2) *Laboratory record screening:* Laboratory records from several hospitals or clinics across Switzerland will be used. Identification will be done based on LDL-c values. After identification, the primary care provider of the potential FH patient will be sent a notification letter or email describing the patient’s high LDL-c level, and the possibility for additional

screening and specialized lipid appointment at one of the study center. The letter or email will contain the information and consent forms of the study.

The recruitment will be done in study centers, which are specialized lipid or cardiology clinics across Switzerland. To be eligible, each site will be required to receive institutional review board approval. Depending on inclusion rate, additional sites will be recruited during the study. Study sites will represent all geographic regions of Switzerland, French, German and Italian, to ensure real-world approaches to FH detection and management.

At the screening visit, V0, the local investigator will identify an eligible participant who is planned for a regular medical visit at the study center, and will send the information and consent forms 2 weeks before the visit (see **Figure 3**).

At the medical visit, V1, the investigators will explain to each participant the nature of the study, its purpose, the procedures involved, the expected duration, the potential risks and benefits and any discomfort it may entail. Each participant will be informed that the participation in the study is voluntary and that he or she may withdraw from the study at any time and that withdrawal of consent will not affect his or her subsequent medical assistance and treatment. The participant will be informed that his or her medical records may be examined by authorised individuals other than their treating physician.

All participants for the study will be provided withinformation and consent forms describing the study and providing sufficient information for participant to make an informed decision about their participation in the study. The formal consent of a participant, using the approved consent form, will be obtained before the participant is submitted to any study procedure.

The consent form will be signed and dated by the investigator or his designee at the same time as the participant sign. A copy of the signed informed consent will be given to the study participant. The consent form will be retained as part of the study records.

There will be no payments given to participants. However, all study procedures will be free-of-charge for participants, including

- genetic tests
- pre and post-counseling visits before and after a genetic test
- additional time taken for study procedures during or outside a medical visit, including central administrative work for cascade screening
- all study visits needed in addition to medical visits.
-

Compensation for a maximal of 10.-CHF per visit will be provided for transport or parking, if requested by the participant based on a proof of payment.

All other medical procedures, laboratory analysis or drug use will be in charge of the participants and their health insurances.

4.3 Study procedures

Standard operation procedures for cascade screening are reported in **Figure 4**. All index cases included in the study will have a genetic test to diagnose FH.

Figure 4

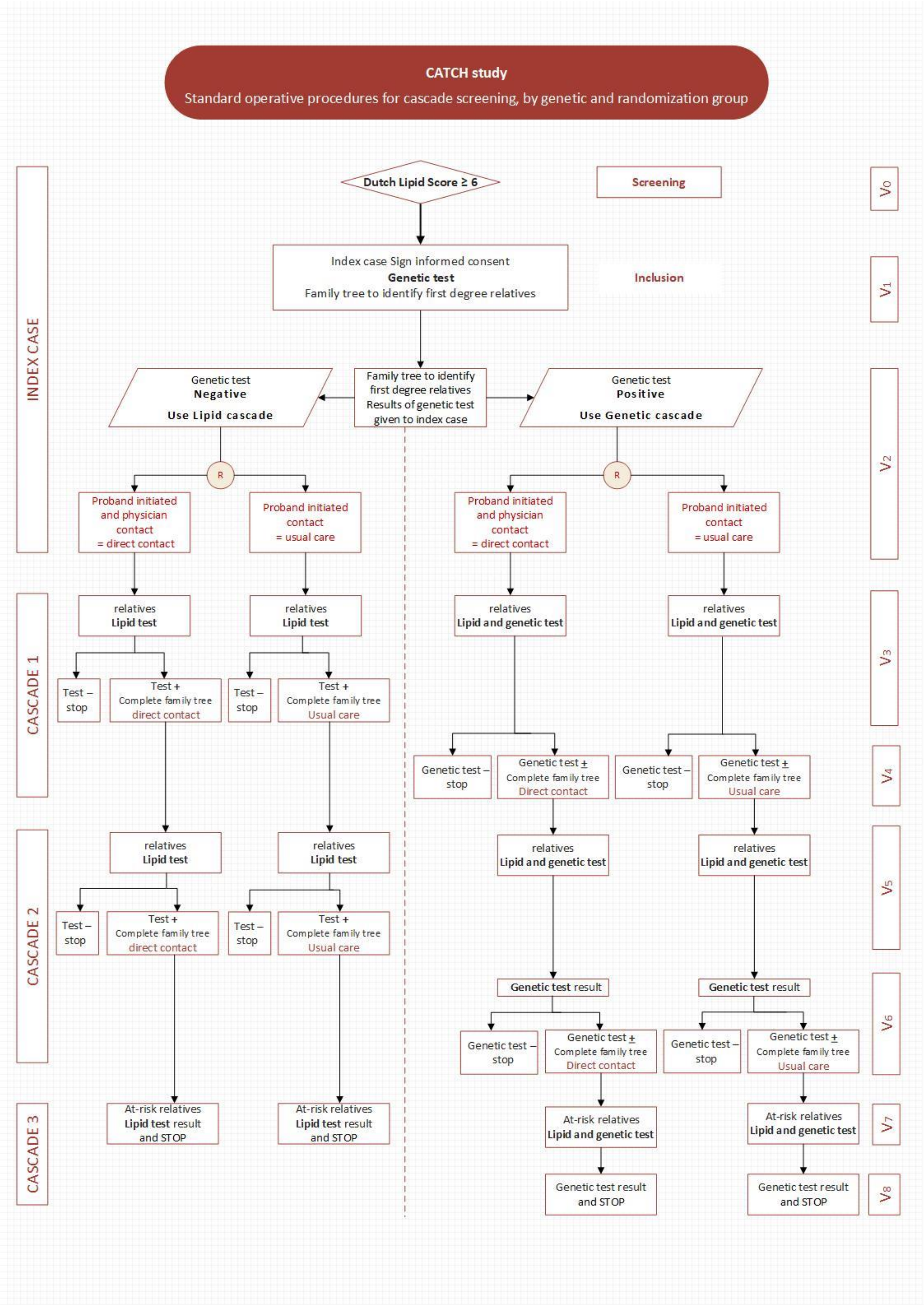
CATCH study
Standard operative procedures for cascade screening, by genetic and randomization group
INDEX CASE
CASCADE 1
CASCADE 2
CASCADE 3
Dutch Lipid Score ≥ 6
Screening
V0
Index case Sign informed consent
Genetic test
Family tree to identify first degree relatives
Inclusion
V1
Genetic test
Negative
Use Lipid cascade
Family tree to identify first degree relatives
Results of genetic test given to index case
Genetic test
Positive
Use Genetic cascade
V2
R
Proband initiated and physician contact = direct contact
Proband initiated contact = usual care
relatives
Lipid test
relatives
Lipid and genetic test
V3
Test – stop
Test + Complete family tree direct contact
Test + Complete family tree Usual care
Genetic test – stop
Genetic test ± Complete family tree Direct contact
Genetic test ± Complete family tree Usual care
V4
V5
Genetic test result
V6
At-risk relatives
Lipid test result and STOP
At-risk relatives
Lipid and genetic test
V7
Genetic test result and STOP
V8

Genetic tests of index cases and relatives

Blood sample of 7.5 mL(2.5 ml for children and adolescents) will be specifically collected and shipped to the central laboratory for genetic analysis and biobank. The genetic testing for FH requires a 7.5 ml EDTA tube of peripheral blood. Genetic tests will be centralized and performed in the accredited medical laboratory of Service de génétique médicale of Hôpital du Valais (HVS) – Institut Central des Hôpitaux (ICH) in Sion. This lab participate regularly to external quality controls for FH genetic analysis. The lab will provide a report for each patient within 2 months. In order to reach the sample size, aproximately 250 index cases will be screened for mutations in *LDLR*, *APOB* and *PCSK9*. The presence/absence of a mutation in the 3 primary genes of FH, namely *LDLR*, *APOB* and PCSK9, will be communicated to the patient by the physician in charge of the patient (see below *Restitution of genetic information*). No other genetic disease will be evaluated by the laboratory. For First-degree relatives of an indexcase with a *positive* mutation, only the specific familial variant will be identified.

Biobank

A biobank will also be created and maintained in the same lab in Sion for further research project on cardiovascular risk. After DNA extraction, the remaining blood is stored at -20°C for at least 2 years. DNA is stored at -80°C for at least 5 years. The freezers are located in the laboratory of the Institut Central des Hôpitaux in Sion; the freezers are locked, with only collaborators of the Service de Génétique médicale having access. The tubes (both blood and DNA) are labelled with the patient's first name, name, date of birth and lab identifier. An Excel-based spreadsheet with all relevant sample information (first name, name, date of birth and lab identifier, result of genetic testing) is stored in the secured environment of the hospital's IT infrastructure. The sheet is password-protected, with only collaborators of the Service de Génétique médicale having access.

Laboratory methods for genetic analyses

For the index case, the lab will use next generation sequencing (NGS), a technique that allows to sequencing several genes of interest in a single reaction. The NGS technology consists of the following steps: DNA extraction, library preparation, NGS sequencing, NGS data analysis, and interpretation.

We will use the library preparation method developed by the company Devyser (kit FH v2). This kit amplifies the coding sequences (i.e. those parts of the gene that finally produce the protein) of *LDLR*, *APOB*, *PCSK9, LDLRAP1, APOE* and *STAP1.* In addition, it amplifies 12 SNPs associated with polygenic FH , as well as 4 SNPs associated with pharmacogenetic effects of statin therapy.[14] The amplified regions are then sequenced on a MiSeq instrument (Illumina). The data generated on the MiSeq – the so-called fastq-files – are uploaded on the DDM platform of SophiaGenetics in a pseudonymised form, i.e. no names and no DOBs are communicated to SophiaGenetics. Of note, the DDM server is located in Switzerland, and the service fulfils the legal requirements regarding data protection. DDM aligns the sequences generated to a reference sequence and identifies all differences between the reference sequence and the patient's sequence. In the next step, the lab analyses all the variants identified in the genes of interest, i.e. *LDLR*, *APOB* and *PCSK9*. The other regions that were also sequenced are not analysed, meaning that eventual variants in these genes are not identified. However, these non-analysed regions can be analysed at a later stage upon the respective request of the physician. Of note, the principle of sequencing

more regions than that are finally analysed is applied since many years in diagnostic genetics labs; the technique is commonly labelled as “virtual panels”. The final stage is the classification of the variants identified in the genes of interest. The classification into the five classes (pathogenic, likely pathogenic, benign, likely benign, and variants of significance/VUS) is done according to the guidelines of the American College of Medical Genetics.[15] Only variants classified as pathogenic, likely pathogenic or VUS will be communicated in the medical report.

For relatives of an index case, only the familial variant identified in the index case will be tested for. This will be done by classical Sanger sequencing.

Differences between the monogenic positive and negative groups

The test used for cascade screening will be different for relatives of index cases with monogenic *positive* FH and for relatives of monogenic *negative* FH index case.

a) *Relatives of a monogenic positive FH index case*
   We will use a genetic and a lipid test to diagnose FH according to guidelines.[5,6] If the patient refuse to perform a genetic test, a lipid test only will be used. If the genetic test is positive, the family tree will be completed with the patient and a second cycle of cascade screening will be performed (see **Figure 2**). If the genetic test is negative, the cascade screening will stop. There will be a maximum of three cycle of identification. For each genetic test done, study center will organize a pre and post-test counselling visit.

b) *Relatives of monogenic negative FH index case*
   We will use a lipid test only to diagnose FH according to guidelines.[5,6] For adults, the diagnosis of FH will be based on established LDL-c criteria available online https://www.nice.org.uk/guidance/cg71/evidence/full-guideline-appendix-f-pdf-241917811.
   - For children from 5 years to 15 years old, diagnosis of FH will be based on a documented untreated/treated LDL-cholesterol of 4.0/3.0 mmol/l or above, or 3.5/3.0 mmol/l or above in case of a family history (see paragraph above for details).

Study centers

The identification and the inclusion of the index case, as well as the pre and post visit counseling for genetic test will be performed by study centers. Study centers will be located in university or peripheral clinics specialized in lipid management. Clinicians involved in the study will be selected based on their expertise to manage lipid abnormalities and are all aware of the genetic characteristics of FH (*see below*). In addition, these clinicians will be trained on how to inform patients about the consequences of genetic tests for themselves and for their families. Study center who will include children are trained to perfom blood test in children and to take care of adolescent according to best-medical practice, with well-trained nursing team.

Restitution of genetic information

FH is an autosomal dominant genetic disease. This means that
- the mutated gene is on a non-sexual chromosome (not the X or Y chromosome).
- the presence of a single mutated gene is enough for the disease to develop.
In practice this leads to the following characteristics:
- the disease affects both men and women

- each member of the family has 50% probability of receiving the genetic mutation

This non-complex mode of inheritance does not require advanced genetic knowledge to counsel families. Therefore, this information will be provided to patients by the physician of the study center. An additional consultation with a geneticist is not required. In case of doubt before the result is given to the patient, the interpretation of identified variants can be discussed with the biologist Dr Von Kanel in Sion.

Genetic analysis will be in charge of the investigator only if shipment of tubes are addressed to the central laboratory accredited for the Study. Similarly, for relatives of an index case with a monogenic mutation for FH, genetic analysis will be in charge of the investigator only if shipment of tubes are addressed to the same central laboratory accredited for the Study.

Study duration

The study duration and the number of visit for each participant will differ according to the protocol (**Table 1**):

Table 1: study duration for each participant

**Participant**	**Study duration in months**	**Number of study visits overall**	**Number of mandatory one-site study visit**
Index case	8	3	1
Relative of a monogenic *postive* FH with postive genetic test	7	3	1
Relative of a mongoenic *postive* FH with negative genetic test	1	2	1
Relative of a monogenic *negative* FH with postive lipid test	6	2	1
Relative of a monogenic *negative* FH with negative lipid test	0	1	1

The inclusion of the 154 index cases is planned over 30 months (**Table 2**). The inclusion of the about 350 relatives is planned over 42 months. The time allowed to complete the three cycles of identification using cascade screening among families will be 12 months.
A 6-month follow-up visit will be performed for each of the 154 index cases, as well as for all relatives with a positive genetic or lipid test.
The total study duration will be 4 years: planned 06/2020 of First-Participant-In, planned 12/2024 of Last-Participant-Out.

Table 2 : Overall study duration

**Year**	**2020**	**2021**	**2022**	**2023**	**2024**
Recruitment of index cases					
Visits of index cases					
Inclusion of relatives					
Visits of relatives					

The schedule of assessments for each type of participant is described in **Table 3a-c**

Table 3a-c: schedule of assessments

a) ***Table 3a**: Schedule of assessments for index cases*

Time (months)	>-1	0	+3	+9
**Visit window (weeks)**	**± 4**	**0**	**± 4**	**± 4**
**Visit (see Figure 2)**	Screening (V0)	Inclusion (V1)	Index case (V2)	Follow-up (V2F)
**Type of contact**	Letter	On site	On site or telephone	On site or telephone
**Written patient information**	+	+		
**Oral and written patient information**		+		
**Written consent**		+		
**Inclusion-/ exclusion criteria**		+		
**eCRF**		+	+	+
**Intervention**			+	
**Self-reported questionnaire baseline**		+		
**Self-reported questionnaire follow-up**				+
**Physical examination**		+		
**Genetic test**		+		
**Laboratory documentation**		+		+
**Qualitative research (sample)**				+

See Appendix 1 for collected variables in each questionnaire/eCRF

b) ***Table 3b:** Schedule of assessments for relatives of the monogenic positive FH group*

Time (months)	0	+2	+8
**Visit window (weeks)**	**0**	**± 4**	**± 4**
**Visit (see Figure 2)**	Cascade (V3 or V5 or V7)	Cascade (V4 or V6 or V8)	Follow-up only if genetic criteria for FH (V4F or V6F or V8F)
**Type of contact**	On site	On site or telephone	On site or telephone
**Oral and written patient information**	+		
**Written consent**	+		
**Inclusion-/ exclusion criteria**	+		
**Intervention**		+	
**eCRF**	+	+	+
**Self-reported questionnaire baseline**	+		

**Self-reported questionnaire follow-up**			+
**Physical examination**	+		
**Genetic test**	+		
**Laboratory documentation**	+		+
**Qualitative research (sample)**			+

See Appendix 1 for collected variables in each questionnaire/eCRF

c) ***Table 3c**: Schedule of assessments for relatives of the monogenic negative FH group*

**Time (months)**	**0**	**+6**
**Visit window (weeks)**	**0**	**± 4**
**Visit (see Figure 2)**	Cascade (V3 or V5 or V7)	Follow-up only if clinical criteria for FH (V3F or V5F or V7F)
**Type of contact**	On site, by telephone if on site not possible	On site or telephone
**Oral and written patient information**	+	
**Written consent**	+	
**Inclusion-/ exclusion criteria**	+	
**eCRF**	+	+
**Intervention**	+	
**Self-reported questionnaire baseline**	+	
**Self-reported questionnaire follow-up**		+
**Physical examination**	+	
**Cholesterol test if not available**	+	
**Laboratory documentation**	+	+
**Qualitative research (sample)**		+

See Appendix 1 for collected variables in each questionnaire/eCRF

Collection of variables

The primary source of information will be the patient's medical record. Baseline data elements to be abstracted and entered include patient demographics, medical history, patient FH history and diagnosis, FH type, family history, physical examination findings including blood pressure and physical signs of lipid accumulation in the tissue, current lipid-lowering therapies and laboratory values. A list of variable collected is found in the **Appendix 1**.

Laboratory documentation

The most recent laboratory measurement will be collected for lipids according to **Appendix 1.**

4.4 Withdrawal and discontinuation

Participants may withdraw from the study at any time at their own request, or they may be withdrawn at any time at the discretion of the investigator for safety or behavioral reasons, or the inability of the subject to comply with the protocol required schedule of study visits or procedures.

Participants who refuse to submit a questionnaire will remain in the study and will continue to be followed for protocol specified follow-up procedures. The only exceptions to this are when a participant specifically withdraws consent for any further contact with him/her or persons previously authorized by subject to provide this information. Medical data and biological material (blood samples, tissues, etc.) collected so far will still be analysed, so as not to compromise the value of the study as a whole. After the analysis, data and biological material will be made anonymous by permanently erasing the code linking to identifiers.

If a participant does not return for a scheduled visit, every effort should be made to contact him, to reschedule the visit, by phone, e-mail, text message, and if necessary, by letter and/or certified mail. All reasonable efforts must be made to locate participants to determine and report their ongoing status. This includes follow-up with persons authorized by the participants.

Lost to follow-up is defined by the inability to reach the participant after a minimum of three documented phone calls, faxes, or emails as well as lack of response by participant to one registered mail letter. Additional attempts to contact the participant may be performed, until the study is completed.
.

5 STATISTICS AND METHODOLOGY

5.1. Statistical analysis plan and sample size calculation

The sample size has been calculated according to the primary outcome, for each of the study groups, monogenic *postive* and *negative* FH. The statistician team of Unisanté (Jerome Pasquier and Valentin Rousson) has estimated the sample size.

The aim of the study is to show that the proportion of people who agree to do the screening is higher in the experimental group than in the control group. Consents and refusals being measured within the families, the observations are not independent. Thus, it would be inappropriate to compare the consent rates of the control and experimental groups using a chi-square test. To account for the effect of clusters, proportions will be compared using a odds ratio that will be estimated using a logistic regression model. The latter will include the group as a fixed effect (the parameter of interest) and the family as a random effect.

For the monogenic *positive* FH group, we calculated that it will be necessary to include 41 families per group to show a statistically significant difference between the two groups with a significance level of 5% and a power of 90%, assuming that the consent rates for the control and interventional groups will be in mean equal to 0.15 respectively 0.3 and will be at 95% in the intervals [0.1,0.2] (range 0.05) respectively [0.1,0.5] (range 0.2) and that the number of contactable relatives will be in mean equal to 4.

For the monogenic *negative* FH group, we calculated that it will be necessary to include 36 families per group to show a statistically significant difference between the two groups with a significance level of 5% and a power of 90%, assuming that the consent rates for the control and interventional groups will be in mean equal to 0.5 respectively 0.7 and will be at 95% in the intervals [0.30,0.70] (range 0.4) respectively [0.46,0.86] (range 0.4) and that the number of contactable relatives will be in mean equal to 4.

These sample sizes were calculated analytically based on the standard errors of the mean proportions and were verified using simulations.

5.2. Handling of missing data and drop-outs

Missing data will be account for with multiple imputation analysis and/or complete case analysis. As it is an effectiveness trial, drop-outs will not be replaced by recruitment of new subjects.

6 REGULATORY ASPECTS AND SAFETY

6.1 Local regulations / Declaration of Helsinki

This study is conducted in compliance with the protocol, the current version of the Declaration of Helsinki, the ICH-GCP, the HRA as well as other locally relevant legal and regulatory requirements.

6.2 (Serious) Adverse Events

An Adverse Event (AE) is any untoward medical occurrence in a patient or a clinical investigation subject which does not necessarily have a causal relationship with the trial procedure. An AE can therefore be any unfavorable or unintended finding, symptom, or disease temporally associated with a trial procedure, whether or not related to it.

A Serious Adverse Event (SAE) (ClinO, Art. 63) is any untoward medical occurrence that

- Results in death or is life-threatening,
- Requires in-patient hospitalisation or prolongation of existing hospitalization,
- Results in persistent or significant disability or incapacity, or
- Causes a congenital anomaly or birth defect

Both Investigator and Sponsor-Investigator make a causality assessment of the event to the trial intervention, (see table below based on the terms given in ICH E2A guidelines). Any event assessed as possibly, probably or definitely related is classified as related to the trial intervention.

Relationship	Description
Definitely	Temporal relationship<br>Improvement after dechallenge*<br>Recurrence after rechallenge<br>(or other proof of drug cause)
Probably	Temporal relationship<br>Improvement after dechallenge<br>No other cause evident
Possibly	Temporal relationship<br>Other cause possible
Unlikely	Any assessable reaction that does not fulfil the above conditions
Not related	Causal relationship can be ruled out
*Improvement after dechallenge only taken into consideration, if applicable to reaction	

Both Investigator and Sponsor-Investigator make a severity assessment of the event as mild, moderate or severe. Mild means the complication is tolerable, moderate means it interferes with daily activities and severe means it renders daily activities impossible.

Reporting of SAEs (see ClinO, Art. 63)

All SAEs are documented and reported immediately (within a maximum of 24 hours) to the main Investigator of the study. If it cannot be excluded that the SAE occurring in Switzerland is attributable to the intervention under investigation, the Investigator reports it to the Ethics Committee via BASEC within 15 days.

If the SAE occurs at one of the study sites, the coordinating Investigator reports the events to the Ethics Committee concerned, within 15 days.

Exemptions from expedited reporting will be done for incident cardiovascular event, because cardiovascular disease is an established result of FH. Therefore, the SAEs that are exempted from expedited reporting are CV death, any MI (fatal and non-fatal); any stroke (fatal and non-fatal); hospitalization for unstable angina; hospitalization for congestive heart failure (CHF); any coronary revascularization procedure; CABG ; any arterial revascularizations.

Follow up of (Serious) Adverse Events

All other medical procedures than those described in the protocol under ch.4.2, including laboratory analysis, medical visits or drug use will be in charge of the participants and their health insurances. This also include follow up procedures of participants terminating the study with reported ongoing (S)AEs.

6.3 (Periodic) safety reporting

An annual safety report (ASR/DSUR) is submitted once a year to the local Ethics Committee by the Investigator (ClinO, Art. 43 Abs). In international multicentric studies the ASR/DSUR contains information from all sites including information from sites outside of Switzerland. The Sponsor-Investigator distributes the ASR/DSUR to all the participating Investigators.

6.4 Radiation

If applicable, please refer to the swissethics template of clinical trials for IMPs and Medical Devices.

6.5 Pregnancy (if applicable)

Reporting of pregnancies will not be necessary.

6.6 Amendments

Substantial changes to the study setup and study organization, the protocol and relevant study documents are submitted to the Ethics Committee for approval before implementation. Under emergency circumstances, deviations from the protocol to protect the rights, safety and well-being of human subjects may proceed without prior approval of the Ethics Committee. Such deviations shall be documented and reported to the Ethics Committee as soon as possible.

Substantial amendments are changes that affect the safety, health, rights and obligations of participants, changes in the protocol that affect study objective(s) or central research topic, changes of study site(s) or of study leader and sponsor (ClinO, Art. 29).

A list of substantial changes is also available on www.swissethics.ch.

A list of all non-substantial amendements will be submitted once a year to the competent EC

together with the ASR.

6.7 (Premature) termination of study

The Sponsor-Investigator may terminate the study prematurely according to certain circumstances:

- Ethical concerns,
- Alterations in accepted clinical practice that make the continuation of the study unwise, or
- Early evidence of harm or benefit of the experimental intervention

Upon regular study termination, the Ethics Committee is notified via BASEC within 90 days (ClinO, Art. 38).

Upon premature study termination or study interruption, the Ethics Committee is notified via BASEC within 15 days (ClinO, Art. 38).

All biological materials and health-related data are anonymised upon end of data analysis.

6.8 Insurance

In the event of study-related damage or injuries, the liability of the institution Unisanté provides compensation, except for claims that arise from misconduct or gross negligence.

7 FURTHER ASPECTS

7.1 Overall ethical considerations

FH is transmitted to family members with a autosomal dominant mode. Therefore, each first degree relative of an index case has a 50% chance of having the disorder. Because FH increase the life-long cardiovascular risk and can be effectively treated with lipid-lowering drugs and appropriate diet, physicians should ideally inform relatives of an index case of their potential cardiovascular risk and their options to reduce it. However, legal protection to guarantee privacy of data do not authorize physicians to directly contact at-risk relatives of an index case. Alternatively, the index case can be counseled to inform his relatives about the risk of FH. However this process can be hampered by inefficient family communication. Therefore, ethical tension exists between motivation to promote health care and interest in maintaining privacy of health information. This study is designed to study the social and scientific value of a cascade screening program for FH in Switzerland. The cascade screening will include adults and children from age 5 years as recommended in international guidelines for FH, to be sure children will receive the full potential benefit of early identification.[16]

This study will use genetic information related only to FH, by examining specifically 3 genes LDLR, APOB, and PCSK9. No other genetic information will be assessed. Thus, no information about an untreatable genetic disease will be provided. The rational to support genetic testing for FH are: 1) individuals with monogenic *positive* FH have an increased risk of cardiovascular disease compared to monogenic *negative* FH patient, 2) the availability and efficacy treatments to lower LDL-c levels and cardiovascular risk, and 3) a potential improved medication compliance in the presence of a genetic diagnosis, and 4) increase performance of cascade screening among relatives.[5]

To anticipate for future research projects, a consent form to perform other genetic analysis in the study biobank will be provided to participants. By contrast to this protocol, these future research

projects will be performed in a fully anonymized database, without any possibility to return to the identification of the participants.

Specific ethical consideration for children

Children and adolescent from 5 to 17 years old can be included as relatives of an index case. However, because of the design of the intervention, children and adolescent can not be included as index case. Cascade screening programme for FH is recommended for children by clinical guidelines.[13] Lifestyle intervention is particularly effective if started early in life, and a lipid-lowering treatment can also be initiated from 10 years old. The confirmation of a genetic mutation for FH will help shared-decision to initiate effective preventive measure currently available to reduce the life-long cardiovascular risk associated with FH. By participating to this study, children and families will have to opportunity to meet expert in cholesterol management. We will adapt the study to children and adolescent and take only 2.5 ml venous blood (instead of 7.5 ml) to perform the genetic test. It is not possible to use capillary blood to perform a genetic test. Study center who will include children are trained to perfom blood test in children and to take care of adolescent according to best-medical practice, with well-trained nursing team.

7.2 Risk-benefit assessment

The risk of the intervention is related to the psychological effects of unsolicited contact by the cascade screening program. This include a breach in the potential “right not to know” leading to anxiety. The stigmatization of genetic information to obtain a life insurance may also be an issue. These risks have been previously studied in qualitative research and can be minimized.[8,9,11] Indeed, many relatives are already aware that high cholesterol or coronary artery disease runs in their family. This information will also be taken into account by insurers regardless of the genetic information received via cascade screening.

Regarding unauthorised data access, the recording of relatives’contact details, in addition to their name, could be seen as a breach of confidentiality. To protect relatives’ confidentiality, we will not record relatives names and adresses in the cascade screening register until they have positively responded to the index case initiated contact.

The potential benefit of cascade screening for FH have been previously described.

8 QUALITY CONTROL AND DATA PROTECTION

8.1 Quality measures

For quality assurance and quality control, the study personnel will be trained on all important study related aspects. There will be planned quality visits and independent data review through an independent Data- or Safety Monitoring Committee provided by the CTU Lausanne.

For quality assurance the sponsor, the Ethics Committee or an independent trial monitor may visit the research sites. Direct access to the source data and all study related files is granted on such occasions. All involved parties keep the participant data strictly confidential.

8.2 Data recording and source data

The data will be recorded with electronic Case Report Form (eCRF) from Redcap®. The CTU Lausanne will perform an audit trail. For each participant a CRF is maintained. CRFs will not identify participants by their name or birth date, but will provide appropriate coded identification.

The primary source of information will be the patient’s medical record. Source data is all information in original records, certified copies of original records of clinical findings, questionnaires, observations, or other recorded activities in a clinical investigation.

8.3 Confidentiality and coding

Trial and participant data will be handled with uttermost discretion and is only accessible to authorised personnel who require the data to fulfill their duties within the scope of the study. On the CRFs and other study specific documents, participants are only identified by a unique participant number.

We will attribute to each participant two random participation codes: the PID and the SID code (see **Figure 5**). The PID code (personal ID) is a personal non-traceable randomly generated alphanumeric code with 6 digits. This PID will be linked to each participant’s personal identifiable data, such as names, year of birth date, address and contact information, geocodes, profession. Those data will allow identification of family links by centers during the study and will be stored in a dedicated REDCap project. Personal identifiable data will be registered only for participants who signed the informed consent forms. Identifiable data of relatives of a participant will not be collected.

The study SID code (code for research data) is also a non-traceable randomly generated alphanumeric code with 6 digits. The SID code will be used for all data that do not identify participants, such as self-reported questionnaire or laboratory results. Those results will be stored in a dedicated REDCap project. At the end of the study, the database containing personal data will be archived offline and only the IT Unisanté team will be able to access it.

In the intervention group, to help an index case to contact his relatives, we will use a dedicated web-based application, called CATCH family tree app, developed by the IT team at Unisanté led by Julien Thabard. Technically this application will consist in a web interface able to generate preregistered email or SMS to be sent to family members by the participant.

As described in chapter 4.3, biological material (whole blood) will be sent for genetic analysis to Dr Thomas Von Kännel, in the accredited medical laboratory of Service de génétique médicale of Hôpital du Valais (HVS) – Institut Central des Hôpitaux (ICH) in Sion. Dr Thomas Von Kännel will guarantee the confidentiality of participant data in his clinical Lab. Biological material is appropriately stored in a restricted area only accessible to the authorised personnel. Biological material in this study is not identified by participant name but by a unique participant number.

Adresse Av. Grand-Champsec 86, 1950 Sion. Tél. 027 603 48 50 | Fax 027 603 48 57

8.4 Retention and destruction of study data and biological material

All study data including biological material are archived for at least 10 years after study termination or premature termination of the study, at the study site and in the central laboratory.

The study data and the biological materials in the Biobank is planned to be re-use for other research projects. These future research projects will be performed in a fully anonymized database, without any possibility to return to the identification of the participants.

Figure 5

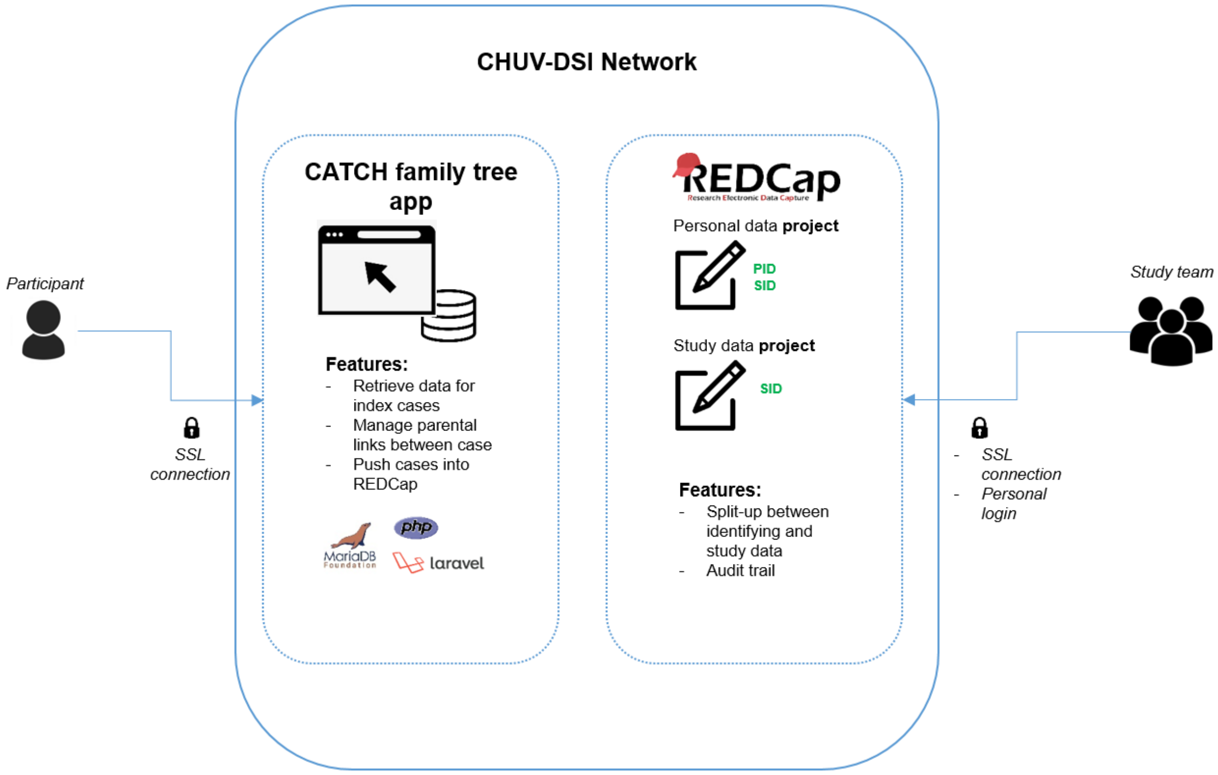

9 MONITORING AND REGISTRATION

The CTU Lausanne will fulfil the monitoring duties, according to their standards, based on collected variables, and will provide a monitoring plan. The CTU of Lausanne is currently writing a monitoring plan (by Laure Vallotton). The monitoring plan will then be submitted to the CER-VD as soon as it is available.

The source data/documents are accessible to monitors and questions will be answered during monitoring.

Registration in a national language in the Swiss National Clinical trial Portal (SNCTP via BASEC) will be done. In addition, the study will be registered in a registry listed in the WHO International Clinical Trials Registry Platform (ICTRP; http://www.who.int/ictrp/en/).

10. FUNDING / PUBLICATION / DECLARATION OF INTEREST

The study is funded by a research grant of the Swiss Heart Foundation. There is no publication policy for the study, and the Swiss Heart Foundation has no role in any aspect of the study. The authors are executive member of the Swiss Atherosclerosis Association (www.gsla.ch) and declare no conflict of interest regarding this protocol. Written agreement between the several study centers will be done.

11 REFERENCES

1. Common Terminology Criteria for Adverse Events (CTCAE) https://www.eortc.be/services/doc/ctc/CTCAE_4.03_2010-06-14_QuickReference_5x7.pdf
2. Declaration of Helsinki https://www.wma.net/policies-post/wma-declaration-of-helsinki-ethical-principles-for-medical-research-involving-human-subjects/
3. Federal Act on Data Protection (FADP) https://www.admin.ch/opc/en/classified-compilation/19920153/index.html
4. Human Research Act (HRA) https://www.admin.ch/opc/de/classified-compilation/20061313/index.html
5. International Conference on Harmonization (ICH) E6(R2) Guideline for Good Clinical Practice http://www.ich.org/fileadmin/Public_Web_Site/ICH_Products/Guidelines/Efficacy/E6/E6_R2__Step_4_2016_1109.pdf
6. International Conference on Harmonization (ICH) E2A Clinical Safety Data Management: Definitions and Standards for Expedited Reporting http://www.ema.europa.eu/docs/en_GB/document_library/Scientific_guideline/2009/09/WC500002749.pdf
7. Ordinance on Clinical Trials in Human Research (ClinO) https://www.admin.ch/opc/de/classified-compilation/20121176/index.html

Expanded references

1. Khera AV, Won HH, Peloso GM, et al. Diagnostic Yield and Clinical Utility of Sequencing Familial Hypercholesterolemia Genes in Patients With Severe Hypercholesterolemia. *Journal of the American College of Cardiology.* 2016;67(22):2578-2589.
2. Miserez AR, Martin FJ, Spirk D. DIAgnosis and Management Of familial hypercholesterolemia in a Nationwide Design (DIAMOND-FH): Prevalence in Switzerland, clinical characteristics and the diagnostic value of clinical scores. *Atherosclerosis.* 2018;277:282-288.
3. Nordestgaard BG, Chapman MJ, Humphries SE, et al. Familial hypercholesterolaemia is underdiagnosed and undertreated in the general population: guidance for clinicians to prevent coronary heart disease: consensus statement of the European Atherosclerosis Society. *Eur Heart J.* 2013;34(45):3478-3490a.
4. Watson KE, Fonarow GC. Closing the Remaining Evidence Gap: Randomized Controlled Trial Data to Support Statin Therapy for Low-Density Lipoprotein >/=190 mg/dL. *Circulation.* 2017;136(20):1892-1894.
5. Sturm AC, Knowles JW, Gidding SS, et al. Clinical Genetic Testing for Familial Hypercholesterolemia: JACC Scientific Expert Panel. *Journal of the American College of Cardiology.* 2018;72(6):662-680.
6. Mach F, Baigent C, Catapano AL, et al. 2019 ESC/EAS Guidelines for the management of dyslipidaemias: lipid modification to reduce cardiovascular risk: The Task Force for the management of dyslipidaemias of the European Society of Cardiology (ESC) and European Atherosclerosis Society (EAS). *European heart journal.* 2019.
7. National Collaborating Centre for Primary Care (UK). Identification and Management of Familial Hypercholesterolaemia (FH) [Internet]. London: Royal College of General Practitioners (UK); 2008 Aug. (NICE Clinical Guidelines, No. 71.) Appendix A, Guidelines scope. Available from: https://www.ncbi.nlm.nih.gov/books/NBK53818/.

8. Hardcastle SJ, Legge E, Laundy CS, et al. Patients' Perceptions and Experiences of Familial Hypercholesterolemia, Cascade Genetic Screening and Treatment. *IntJ Behav Med.* 2015;22(1):92-100.
9. Hallowell N, Jenkins N, Douglas M, et al. A qualitative study of patients' perceptions of the value of molecular diagnosis for familial hypercholesterolemia (FH). *J Community Genet.* 2017;8(1):45-52.
10. Knowles JW, Rader DJ, Khoury MJ. Cascade Screening for Familial Hypercholesterolemia and the Use of Genetic Testing. *JAMA.* 2017;318(4):381-382.
11. Arar NH, Hazuda H, Steinbach R, Arar MY, Abboud HE. Ethical issues associated with conducting genetic family studies of complex disease. *Ann Epidemiol.* 2005;15(9):712-719.
12. Newson AJ, Humphries SE. Cascade testing in familial hypercholesterolaemia: how should family members be contacted? *European journal of human genetics : EJHG.* 2005;13(4):401-408.
13. Wiegman A, Gidding SS, Watts GF, et al. Familial hypercholesterolaemia in children and adolescents: gaining decades of life by optimizing detection and treatment. *Eur Heart J.* 2015;36(36):2425-2437.
14. Talmud PJ, Shah S, Whittall R, et al. Use of low-density lipoprotein cholesterol gene score to distinguish patients with polygenic and monogenic familial hypercholesterolaemia: a case-control study. *Lancet.* 2013;381(9874):1293-1301.
15. Richards S, Aziz N, Bale S, et al. Standards and guidelines for the interpretation of sequence variants: a joint consensus recommendation of the American College of Medical Genetics and Genomics and the Association for Molecular Pathology. *Genetics in Medicine.* 2015;17(5):405-423.
16. Ramaswami U, Futema M, Bogsrud MP, et al. Comparison of the characteristics at diagnosis and treatment of children with heterozygous familial hypercholesterolaemia (FH) from eight European countries. *Atherosclerosis.* 2020;292:178-187.

Appendix 1: List of collected variables

See Questionnaires and eCRFs

Appendix 2: Dutch Lipid Clinic Network score (DLNC) score

Kriterien für die klinische Diagnose der HeFH gemäss Dutch Lipid Clinic Network
Der Algorithmus gilt nur für Erwachsene

Kategorie	Kriterien*	Score
**Familien-anamnese**	Verwandter 1. Grades mit vorzeitiger KHK[1] und/oder Verwandter 1. Grades mit LDL-C ≥5 mmol/l	1
	Verwandter 1. Grades mit Sehnenxanthomen und/oder Arcus lipoides corneae und/oder Kinder <18 J. mit LDL-C >95. Perzentile nach Alter, Geschlecht und Land	2
**Persönliche Anamnese**	Vorzeitige KHK[1]	2
	Vorzeitige cerebrale/periphere Gefässkrankheit[1]	1
**Körperliche Untersuchung**	Sehnenxanthome	6
	Arcus lipoides corneae unter 45 J.	4
**LDL-C (mmol/l)**	>8.5	8
	6.5–8.4	5
	5.0–6.4	3
	4.0–4.9	1
**Genetische Tests**	Nachweis kausaler Mutationen in den Genen LDLR, ApoB oder PCSK9	8
**TOTAL**	**Summe der Punktwerte***	

**Bewertung**	■ Definitive FH	>8
	■ Wahrscheinliche FH	6–8
	■ Mögliche FH	3–5
	■ Keine Diagnose	<3

[1] Mann <55 J., Frau <60 J.

*Benutzung des Algorithmus: pro Kategorie nur einen, nämlich den höchsten, Score verwenden. Zum Beispiel, wenn KHK und Sehnenxanthome in der Familiengeschichte vorkommen, nur den Score 2 verwenden. Wenn nur Personen mit erhöhtem LDL-Cholesterin und frühzeitiger KHK vorkommen, jedoch niemand mit Xanthomen und keine Kinder mit Hypercholesterinämie, nur den Score 1 verwenden.
