# Supplementary material for: Genetic Cascade Screening for Familial Hypercholesterolemia: A Randomized Clinical Trial
Source: JAMA Netw Open. 2026 Apr 13;9(4):e266100. doi: 10.1001/jamanetworkopen.2026.6100 (PMC13077512; doi:10.1001/jamanetworkopen.2026.6100)
Supplement: Supplement 2. — eMethods 1. Web-communication platform eMethods 2. Descriptive variables eTable 1. Prepared email and text message integrated in the web-communication platform. eTable 2. Baseline characteristics of relatives who participated to genetic cascade screening, by study group. eTable 3. Uptake of genetic cascade screening of familial hypercholesterolemia stratified by physical activity level of the referent. eTable 4. Uptake of genetic cascade screening and detection of genetic familial hypercholesterolemia comparing users and non-users of the web-based communication platform. eTable 5. Baseline characteristics of relatives having participated to genetic cascade screening within 6 months of referral, classified by result of genetic test. eFigure 1. Procedures and counselling for genetic cascade screening, according to study group. eFigure 2. Generating Family Trees from the Participant’s Electronic File eFigure 3. Information flow in the implementation intervention group eFigure 4. Content of the web-based platform to invite relatives eFigure 5. 6-month participation probability of relatives by type of relative and by study arm eFigure 6. 6-month participation probability of relatives by screening cycle and by study arm [file jamanetwopen-e266100-s002.pdf]

Supplemental Online Content

Nanchen D, Chaouch A, Scuderi R, et al. Genetic cascade screening for familial hypercholesterolemia: A randomized clinical trial. *JAMA Netw Open or JAMA Health Forum*. 2026;9(4):e266100. doi:10.1001/jamanetworkopen.2026.6100

eMethods 1. Web-communication platform

eMethods 2. Descriptive variables

eTable 1. Prepared email and text message integrated in the web-communication platform.

eTable 2. Baseline characteristics of relatives who participated to genetic cascade screening, by study group.

eTable 3. Uptake of genetic cascade screening of familial hypercholesterolemia stratified by physical activity level of the referent.

eTable 4. Uptake of genetic cascade screening and detection of genetic familial hypercholesterolemia comparing users and non-users of the web-based communication platform.

eTable 5. Baseline characteristics of relatives having participated to genetic cascade screening within 6 months of referral, classified by result of genetic test.

eFigure 1. Procedures and counselling for genetic cascade screening, according to study group.

eFigure 2. Generating Family Trees from the Participant's Electronic File

eFigure 3. Information flow in the implementation intervention group

eFigure 4. Content of the web-based platform to invite relatives

eFigure 5. 6-month participation probability of relatives by type of relative and by study arm

eFigure 6. 6-month participation probability of relatives by screening cycle and by study arm

This supplemental material has been provided by the authors to give readers additional information about their work.

© 2026 Nanchen D et al. *JAMA Network Open*.

eMethods 1: Web-communication platform

The web-based platform was developed using Laravel, a modern PHP framework known for its robustness and scalability. All personal and research data were securely stored in REDCap, a widely used data capture system for clinical research. The two systems were integrated through a secure API, enabling seamless data exchange. Custom developments were implemented within REDCap to support site-specific workflows, including automated email notifications, participant tracking, appointment reminders, and the generation of laboratory labels. These enhancements were designed to streamline operations across participating centers and reduce administrative burden.

© 2026 Nanchen D et al. *JAMA Network Open*.

eMethods 2: ***Descriptive variables***

Socioeconomic status was evaluated based on educational attainment, living arrangement, marital status, and employment status. Smoking status was categorized as never, former, or current. Frequent alcohol use was defined as the consumption of 1 unit three or more times per week for the past six months. Adherence to the Mediterranean diet was assessed using the 14-item Mediterranean Diet Adherence Screener (MEDA) questionnaire. Healthy physical activity level was defined as ≥600 Metabolic Equivalent of Task (MET)-minutes/week using the International Physical Activity Questionnaire (IPAQ). Clinical comorbidities included hypertension, diabetes mellitus, and pre-existing cardiovascular disease, each defined using standard diagnostic or treatment criteria. Lipid profiles (total cholesterol, LDL cholesterol, high-density lipoprotein (HDL) cholesterol, and triglycerides) were reported for both the highest recorded and most recent values. Clinical examination findings such as xanthomas, xanthelasmas, corneal arcus, blood pressure, and body mass index were also assessed. Medication use was recorded for lipid-lowering therapies, aspirin, oral anticoagulants, and antidepressants.

© 2026 Nanchen D et al. *JAMA Network Open*.

eTable 1: prepared email and text message integrated in the web-communication platform

Text message	Email
(name of participant) has written a message for you as part of a Swiss familial hypercholesterolemia screening study:<br>I have taken a genetic test which identified the mutation responsible for my high cholesterol levels. Together with my doctor, I recommend that you also undergo the genetic test, even if your cholesterol levels are normal. The test can be done free of charge at several centers across Switzerland. Click on this secure link to receive more information or to schedule an appointment. Here is our family code: fc-2e1ee90c.	I took a genetic test that identified the mutation responsible for my high cholesterol levels. I'm sharing this with you because each relative has a 50% chance of having the same mutation (a one in two risk). Current treatments for hypercholesterolemia are very safe and highly effective.<br>Together with my doctor, I recommend that you also take the genetic test, even if your cholesterol levels are normal. You can take this test free of charge at several centers in Switzerland. Click on this secure link for more information or to make an appointment. Our family code is: :familycode. Hotline: catch@unisante.ch

© 2026 Nanchen D et al. *JAMA Network Open*.

eTable 2: Baseline characteristics of relatives who participated to genetic cascade screening, by study arm (N=99)

	Control arm (N=32)	Implementation intervention arm (N=67)	p-value
Demographics			
Age, years	35.9 [5.2, 75.7]	27.7 [5.8, 86.1]	0.55
Female	21 (65.6)	36 (53.7)	0.33
Male	11 (34.4)	31 (46.3)	0.33
Socio-economic status			
Higher education[1], n=88	11 (37.9)	23 (39.0)	0.93
Living alone, n=89	4 (13.3)	9 (15.3)	0.87
Married/registered partnership	12 (37.5)	22 (32.8)	0.63
Unemployed/retired, n=88	14 (48.3)	20 (33.9)	0.27
Lifestyle			
Smoking status, n=89			0.97
Never	15 (51.7)	33 (55.0)	
Former	7 (24.1)	14 (23.3)	
Current	7 (24.1)	13 (21.7)	
Frequent alcohol consumption[2], n=88	6 (20.7)	7 (11.9)	0.29
Higher adherence to Mediterranean diet[3], n=81	12 (41.4)	29 (55.8)	0.24
Healthy physical activity[4], n=95	27 (90.0)	43 (66.2)	0.08
Comorbities			
Hypertension[5], n=95	2 (6.2)	4 (6.3)	1.00
Diabetes[6], n=95	1 (3.1)	1 (1.6)	0.78
Cardiovascular diseases[7]	4 (12.5)	6 (9.0)	0.61
Highest recorded lipid parameters			
Total cholesterol, mmol/l, n=41	6.0 [3.5, 11.1]	7.5 [2.7, 9.7]	0.25
LDL cholesterol, mmol/l, n=40	3.9 [2.8, 9.1]	5.7 [1.5, 7.8]	0.33
HDL cholesterol, mmol/l, n=38	1.4 [1.0, 2.4]	1.4 [0.8, 2.2]	0.71
Triglycerides, mmol/l, n=39	1.0 [0.5, 1.8]	1.0 [0.6, 2.3]	0.38
Most recent lipid parameters			
Total cholesterol, mmol/l, n=76	5.8 [2.9, 8.6]	5.4 [2.4, 9.2]	0.53
LDL cholesterol, mmol/l, n=76	4.4 [1.0, 7.3]	3.4 [0.7, 7.1]	0.20
HDL cholesterol, mmol/l, n=75	1.7 [1.0, 2.4]	1.5 [0.8, 3.8]	0.11
Triglycerides, mmol/l, n=76	0.9 [0.4, 2.4]	1.0 [0.5, 5.0]	0.39
Clinical examination			
Xanthomas or xanthelasmas	3 (9.4)	2 (3.0)	0.35
Corneal arcus, n=97	3 (9.4)	1 (1.5)	0.05
Systolic blood pressure, mm Hg, n=86	124.0 [88.0, 156.0]	121.0 [84.0, 171.0]	0.90
Diastolic blood pressure, mm Hg, n=86	76.0 [46.0, 98.0]	73.0 [48.0, 102.0]	0.44

© 2026 Nanchen D et al. *JAMA Network Open*.

	Control arm (N=32)	Implementation intervention arm (N=67)	p-value
BMI, kg/m2, n=84	23.0 [14.0, 31.0]	24.0 [14.0, 35.0]	0.46
Medication			
Lipid lowering drugs, n=88	10 (33.3)	20 (34.5)	0.91
Statins, n=86	9 (31.0)	13 (22.8)	0.35
Ezetimibe, n=86	2 (7.1)	4 (6.9)	0.98
PCSK9 inhibitors, n=86	2 (7.1)	2 (3.4)	0.61
Aspirin, n=87	4 (13.8)	6 (10.3)	0.66
Oral anticoagulants, n=85	0 (0.0)	0 (0.0)	
Antidepressants, n=87	1 (3.4)	2 (3.4)	1.00
Genetics			
Pathogenic variant in the LDLR gene	9 (28.1)	25 (37.3)	0.59

Data are given as number (percentage) or median (range). Categorical variables were compared using Chi-square or Fisher exact tests. Continuous variables were compared using Kruskal-Wallis tests. The null distribution of these test statistics was built using approximate permutation tests (10000 permutations at the family level).

[1]Defined as a high school or university graduation or higher
[2]Defined as three or more time per week since 6 months
[3]Defined as adherence to Mediterranean diet above median according to 14-item MEDAS questionnaire
[4]Defined as 600 MET-minutes/week or more according to IPAQ questionnaire
[5]Defined as physician diagnosed, office systolic blood pressure ≥140 mmHg or diastolic blood pressure ≥90 mmHg or use of blood pressure lowering drugs
[6]Defined as physician diagnosed or use of antihyperglycemic medication or insulin
[7]Defined as coronary heart disease, ischemic cerebrovascular disease or peripheral artery disease

Abbreviations: CVD, cardiovascular disease; LDL, low-density lipoprotein; HDL, high-density lipoprotein; NA, not available; MET, Metabolic Equivalent of Task; IPAQ, International Physical Activity Questionnaire; MEDAS, Mediterranean Diet Adherence Screener ; LDLR, Low-Density Lipoprotein Receptor; APOB, Apolipoprotein B; PCSK9, Proprotein Convertase Subtilisin/Kexin Type 9.

© 2026 Nanchen D et al. *JAMA Network Open.*

eTable 3: Uptake of genetic cascade screening of familial hypercholesterolemia stratified by physical activity level of the referent.

	Eligible relatives	Tested relatives	Raw participation (%)	Estimated participation (%) (95% CI)	OR (95% CI)	p-value
**6-month participation probability of relatives whose referent practice healthy physical activity**						
Overall	274	82	29.9	27.4 (20.3 - 35.8)		
Non-user group	108	20	18.5	16.7 (8.9 - 29.3)		
Web-platform-user group	166	62	37.3	35.2 (25.5 - 46.3)	2.70 (1.14 - 6.37)	0.02
**6-month participation probability of relatives whose referent do not practice healthy physical activity**						
Overall	78	17	21.8	19.6 (11.0 - 32.5)		
Non-user group	54	12	22.2	20.4 (10.4 - 36.2)		
Web-platform-user group	24	5	20.8	17.8 (5.5 - 44.5)	0.84 (0.18 - 3.92)	0.83

p-value for interaction between physical activity of the referent and the study group: 0.15

Abbreviations: CI, confidence interval; OR, odds ratio.

© 2026 Nanchen D et al. *JAMA Network Open*.

eTable 4: Uptake of genetic cascade screening and detection of genetic familial hypercholesterolemia comparing users and non-users of the web-based communication platform.

	Index cases	Eligible relatives	Tested relatives	Raw participation (%)	Estimated participation (%) (95% CI)	OR (95% CI)	p-value
**6-month participation probability of relatives**							
Overall	87	359	99	27.6	23.7 (17.9 - 30.6)		
Non-user group	66	282	62	22.0	18.9 (13.4 - 25.9)		
Web-platform-user group	21	77	37	48.1	43.6 (28.5 - 60.0)	3.32 (1.53 - 7.18)	0.002
**6-month detection probability of relatives with positive genetic test**							
Overall	87	359	55	15.3	12.5 (8.9 - 17.3)		
Non-user group	66	282	36	12.8	10.4 (6.7 - 15.6)		
Web-platform-user group	21	77	19	24.7	21.3 (13.1 - 32.7)	2.34 (1.11 - 4.97)	0.03

Denominator = number of eligible relatives

Abbreviations: CI, confidence interval; OR, odds ratio.

© 2026 Nanchen D et al. *JAMA Network Open*.

eTable 5: Baseline characteristics of relatives having participated to genetic cascade screening within 6 months of referral, classified by result of genetic test (N=99).

	Negative relatives (N=44)	Positive relatives (N=55)	p-value
Demographics			
Age, years	25.7 [5.2, 86.1]	42.0 [5.8, 79.4]	0.02
Female	26 (59.1)	31 (56.4)	0.70
Male	18 (40.9)	24 (43.6)	0.70
Socio-economic status			
Higher education[1], n=88	15 (38.5)	19 (38.8)	0.98
Living alone, n=89	4 (10.3)	9 (18.0)	0.32
Married/registered partnership	14 (31.8)	20 (36.4)	0.66
Unemployed/retired, n=88	20 (51.3)	14 (28.6)	0.37
Lifestyle			
Smoking status, n=89			0.05
Never	28 (71.8)	20 (40.0)	
Former	5 (12.8)	16 (32.0)	
Current	6 (15.4)	14 (28.0)	
Frequent alcohol consumption[2], n=88	5 (12.8)	8 (16.3)	0.65
Higher adherence to Mediterranean diet[3], n=81	16 (43.2)	25 (56.8)	0.49
Healthy physical activity[4], n=95	32 (74.4)	38 (73.1)	0.87
Comorbities			
Hypertension[5], n=95	2 (4.8)	4 (7.5)	0.61
Diabetes[6], n=95	0 (0.0)	2 (3.8)	0.33
Cardiovascular diseases[7]	1 (2.3)	9 (16.4)	0.04
Highest recorded lipid parameters			
Total cholesterol, mmol/l, n=41	5.2 [2.7, 8.6]	7.8 [4.6, 11.1]	0.002
LDL cholesterol, mmol/l, n=40	3.2 [1.5, 6.6]	5.8 [2.9, 9.1]	0.005
HDL cholesterol, mmol/l, n=38	1.4 [0.8, 2.4]	1.4 [1.0, 2.2]	0.89
Triglycerides, mmol/l, n=39	0.8 [0.5, 2.3]	1.1 [0.6, 2.2]	0.02
Most recent lipid parameters			
Total cholesterol, mmol/l, n=76	4.5 [2.4, 8.6]	6.5 [2.9, 9.2]	<0.001
LDL cholesterol, mmol/l, n=76	2.6 [0.7, 6.3]	4.4 [1.0, 7.3]	<0.001
HDL cholesterol, mmol/l, n=75	1.6 [0.9, 3.8]	1.6 [0.8, 2.2]	0.41
Triglycerides, mmol/l, n=76	0.9 [0.4, 5.0]	1.0 [0.4, 4.3]	0.58
Clinical examination			
Xanthomas or xanthelasmas	0 (0.0)	5 (9.1)	0.12
Corneal arcus, n=97	0 (0.0)	4 (7.5)	0.12
Systolic blood pressure, mm Hg, n=86	115.0 [88.0, 161.0]	124.0 [84.0, 171.0]	0.05

© 2026 Nanchen D et al. *JAMA Network Open.*

	Negative relatives (N=44)	Positive relatives (N=55)	p-value
Diastolic blood pressure, mm Hg, n=86	72.0 [46.0, 102.0]	76.0 [57.0, 96.0]	0.44
BMI, kg/m2, n=84	22.0 [14.0, 35.0]	25.0 [14.0, 35.0]	0.13
Medication			
Lipid lowering drugs, n=88	3 (7.9)	27 (54.0)	<0.001
Statins, n=86	2 (5.4)	20 (40.8)	<0.001
Ezetimibe, n=86	0 (0.0)	6 (12.5)	0.14
PCSK9 inhibitors, n=86	0 (0.0)	4 (8.3)	0.13
Aspirin, n=87	1 (2.6)	9 (18.4)	0.02
Oral anticoagulants, n=85	0 (0.0)	0 (0.0)	
Antidepressants, n=87	1 (2.6)	2 (4.1)	0.99
Genetics			
Pathogenic variant in the LDLR gene	0 (0.0)	34 (61.8)	<0.001

Data are given as number (percentage) or median (range). Categorical variables were compared using Chi-square or Fisher exact tests. Continuous variables were compared using Kruskal-Wallis tests. The null distribution of these test statistics was built using approximate permutation tests (10000 permutations of subjects within families).

[1]Defined as a high school or university graduation or higher
[2]Defined as three or more time per week since 6 months
[3]Defined as adherence to Mediterranean diet above median according to 14-item MEDAS questionnaire
[4]Defined as 600 MET-minutes/week or more according to IPAQ questionnaire
[5]Defined as physician diagnosed, office systolic blood pressure ≥140 mmHg or diastolic blood pressure ≥90 mmHg or use of blood pressure lowering drugs
[6]Defined as physician diagnosed or use of antihyperglycemic medication or insulin
[7]Defined as coronary heart disease, ischemic cerebrovascular disease or peripheral artery disease
Abbreviations: CVD, cardiovascular disease; LDL, low-density lipoprotein; HDL, high-density lipoprotein; NA, not available; MET, Metabolic Equivalent of Task; IPAQ, International Physical Activity Questionnaire; MEDAS, Mediterranean Diet Adherence Screener ; LDLR, Low-Density Lipoprotein Receptor; APOB, Apolipoprotein B; PCSK9, Proprotein Convertase Subtilisin/Kexin Type 9.

© 2026 Nanchen D et al. *JAMA Network Open.*

eFigure 1: Procedures and counselling for genetic cascade screening, according to study group.

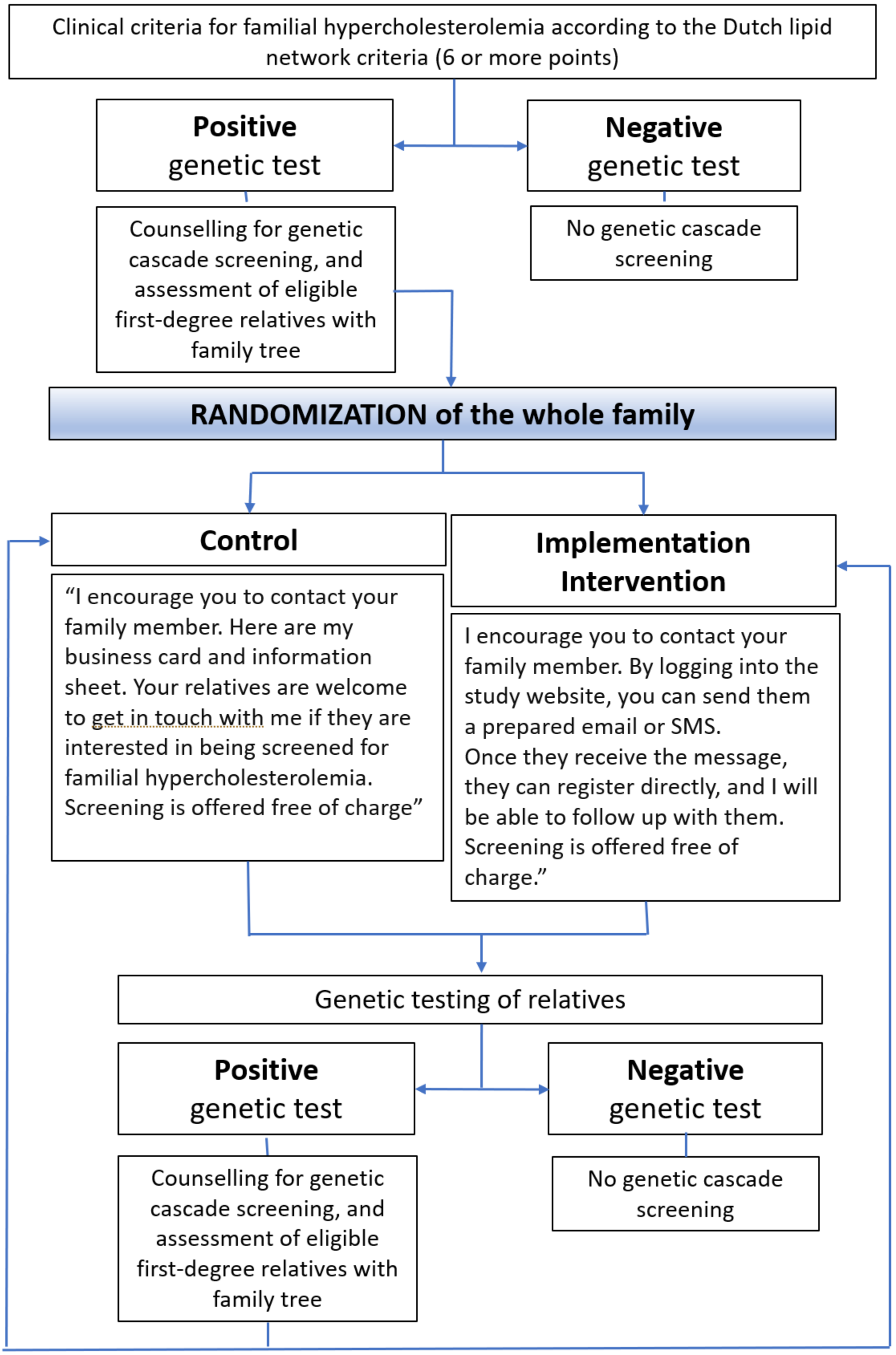

© 2026 Nanchen D et al. *JAMA Network Open*.

eFigure 2: Generating Family Trees from the Participant's Electronic File

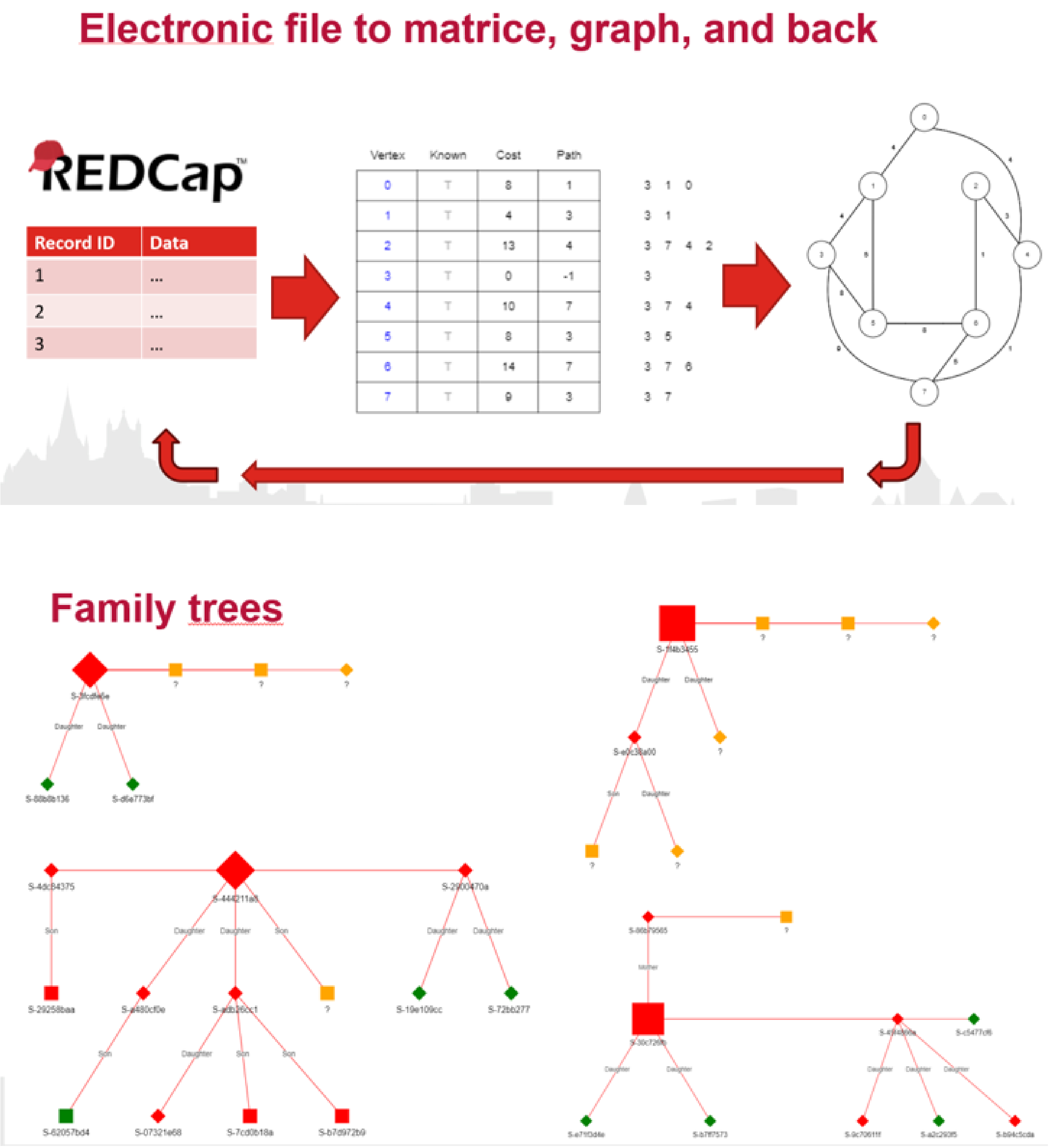

The figure illustrates the process of transforming electronic participant data from REDCap® into matrix and graph structures to automate the generation of family trees. In the upper panel, data from the electronic file is converted into a graph representation and back, enabling computational analysis. The lower panel shows examples of automatically generated family trees.

© 2026 Nanchen D et al. *JAMA Network Open*.

Legend: Colored nodes represent individuals:

- big node for index cases, small nodes for relative,
- red for positive genetic test, green for negative genetic test, orange for undetected or uncontacted relatives,
- square form men, and diamond for women.

© 2026 Nanchen D et al. *JAMA Network Open*.

eFigure 3: Information flow in the implementation intervention group

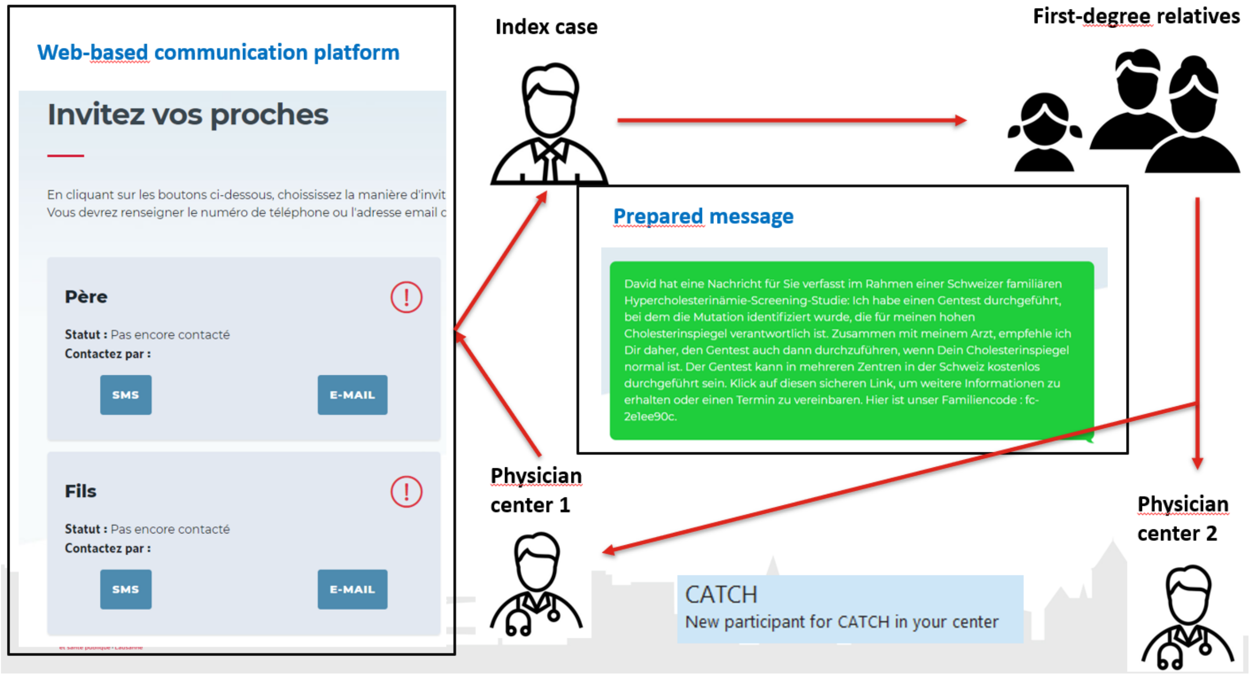

1) **Counselling with Index Case**
   Index cases are counselled by the study team to contact their relatives with the help of a web-based communication platform.
2) **Access to the platorm**
   Index cases receive a unique login and password to access the secure study portal. This portal allows them to invite their relatives to participate in the screening process.
3) **Invitation of Relatives**
   Through the portal, index cases can send pre-prepared messages (email and SMS) to their relatives. These messages contain a personal identification code and instructions for accessing the portal.
4) **Relative Registration**
   Invited relatives who choose to participate use the code to register via the study portal. They provide consent and basic information through a secure online form, and they are given the option to select the screening center closest to their residence.
5) **Follow-Up by Study Team**
   Once registered, relatives are contacted directly by the chosen study team for further screening procedures, including genetic testing.

© 2026 Nanchen D et al. *JAMA Network Open*.

eFigure 4: Content of the web-based platform to invite relatives

1) Inital Email to encourage family contact in the intervention group

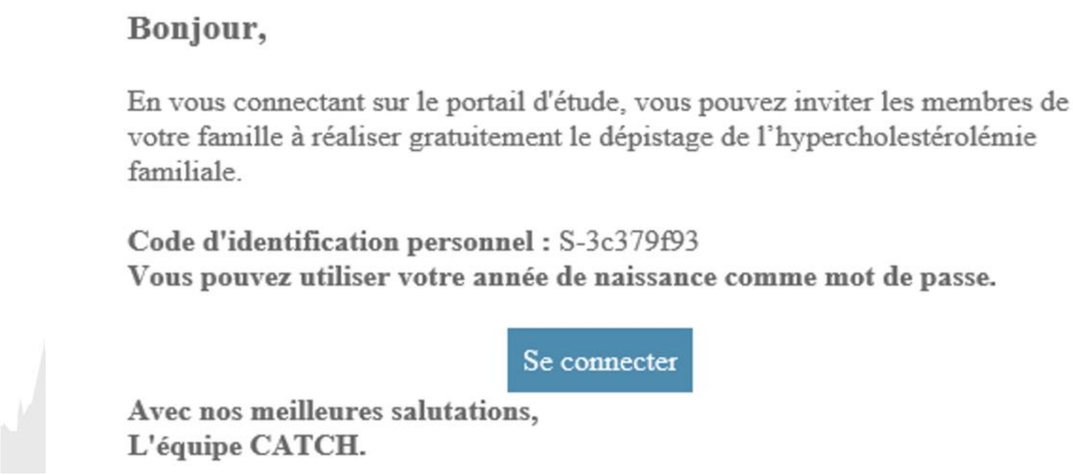

2) Participant portal activated to send prepared email or SMS

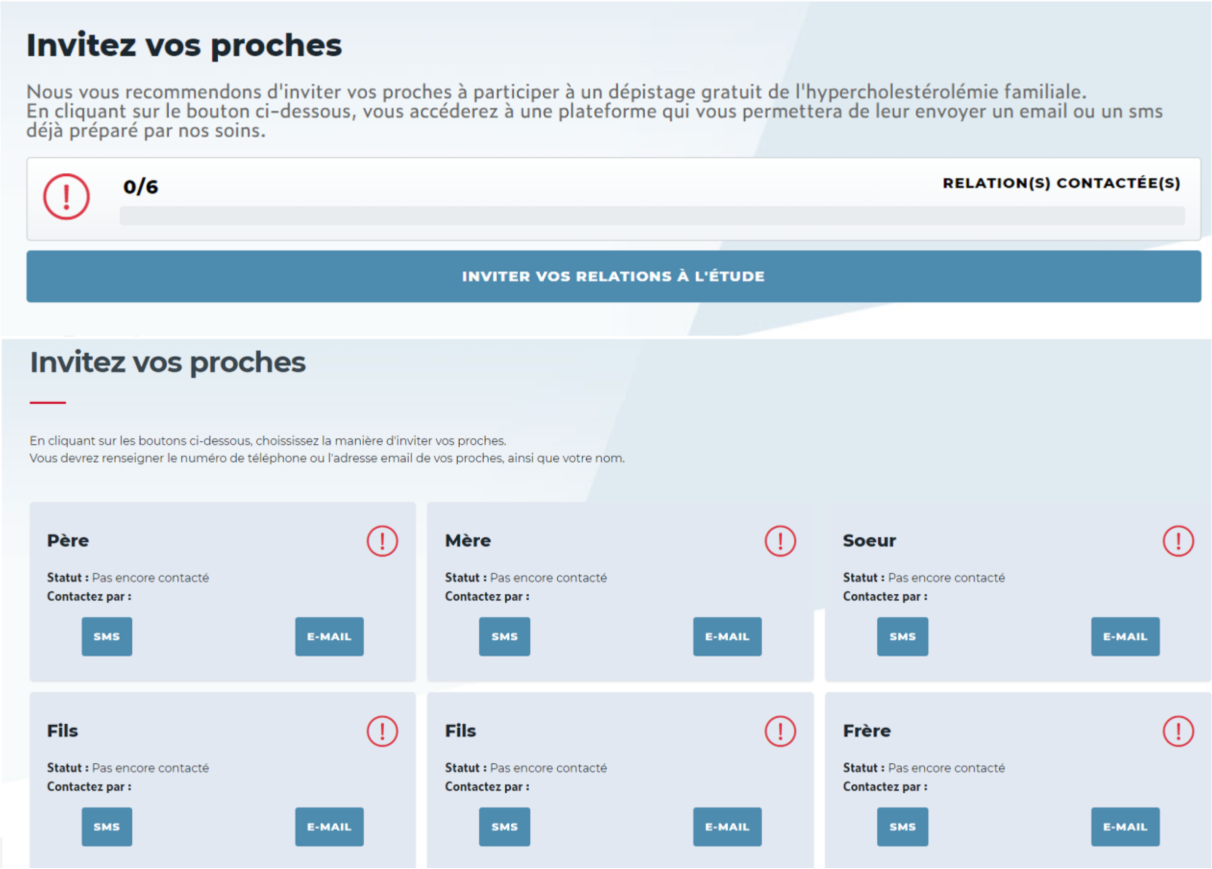

© 2026 Nanchen D et al. *JAMA Network Open*.

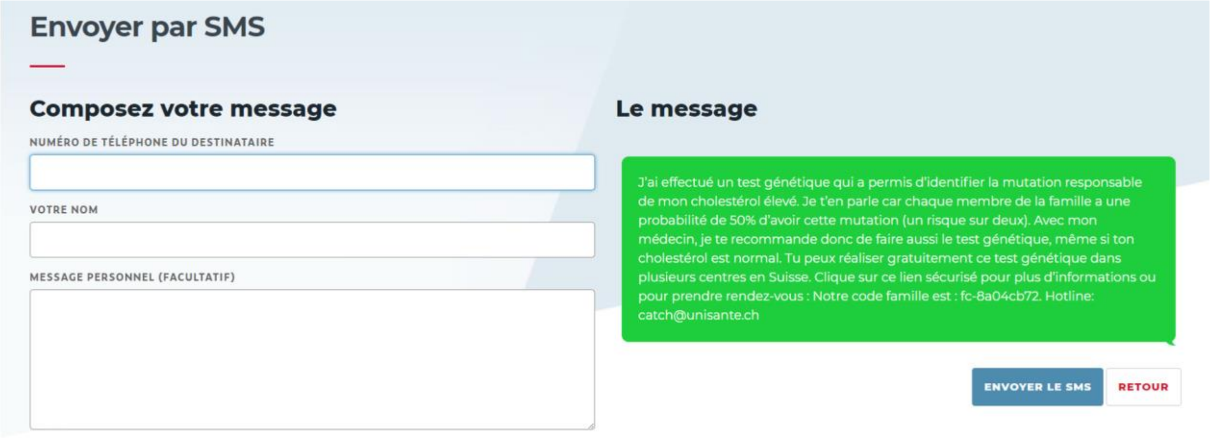

3) Relatives can access the participant portal through the email or SMS invitation

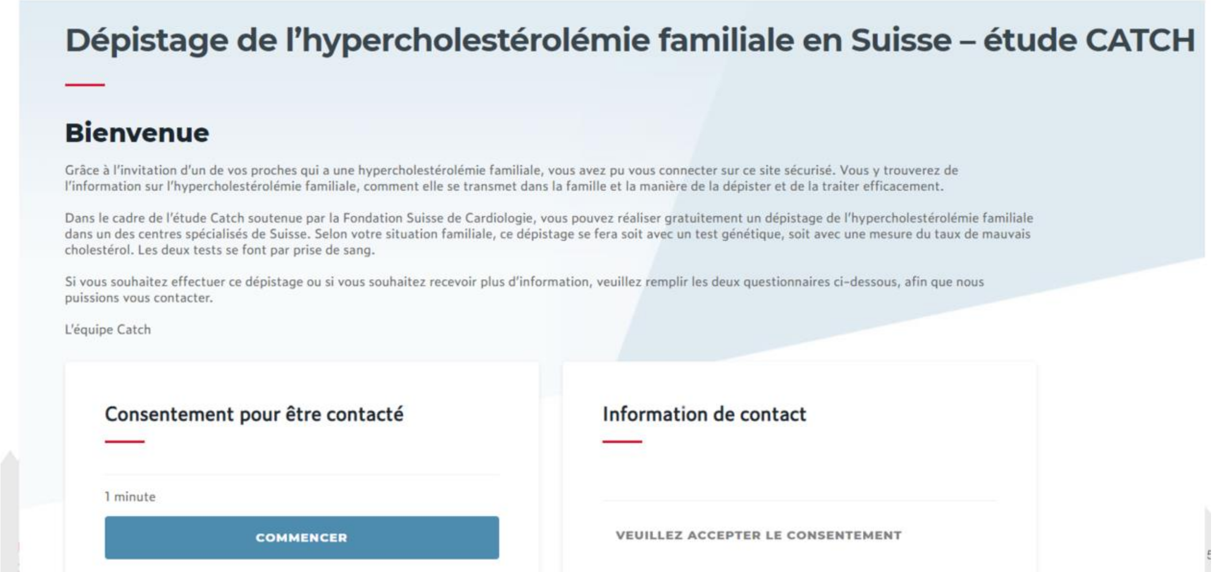

4) Email sent to study site investigator

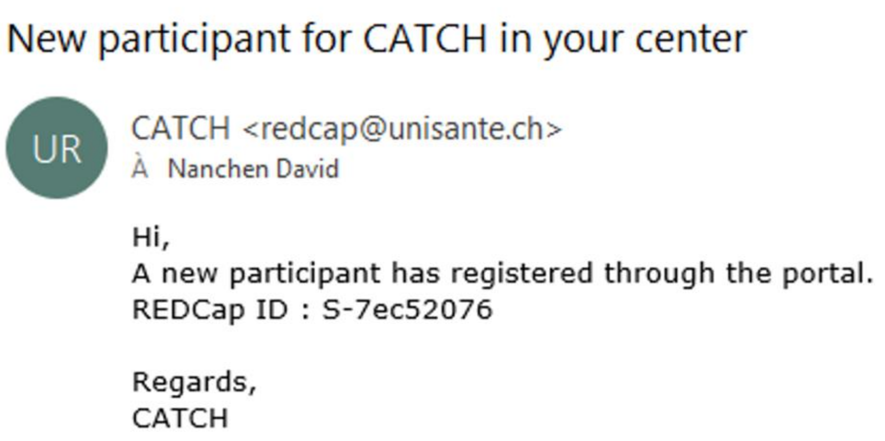

© 2026 Nanchen D et al. *JAMA Network Open*.

eFigure 5: 6-month participation probability of relatives by type of relative and by study arm

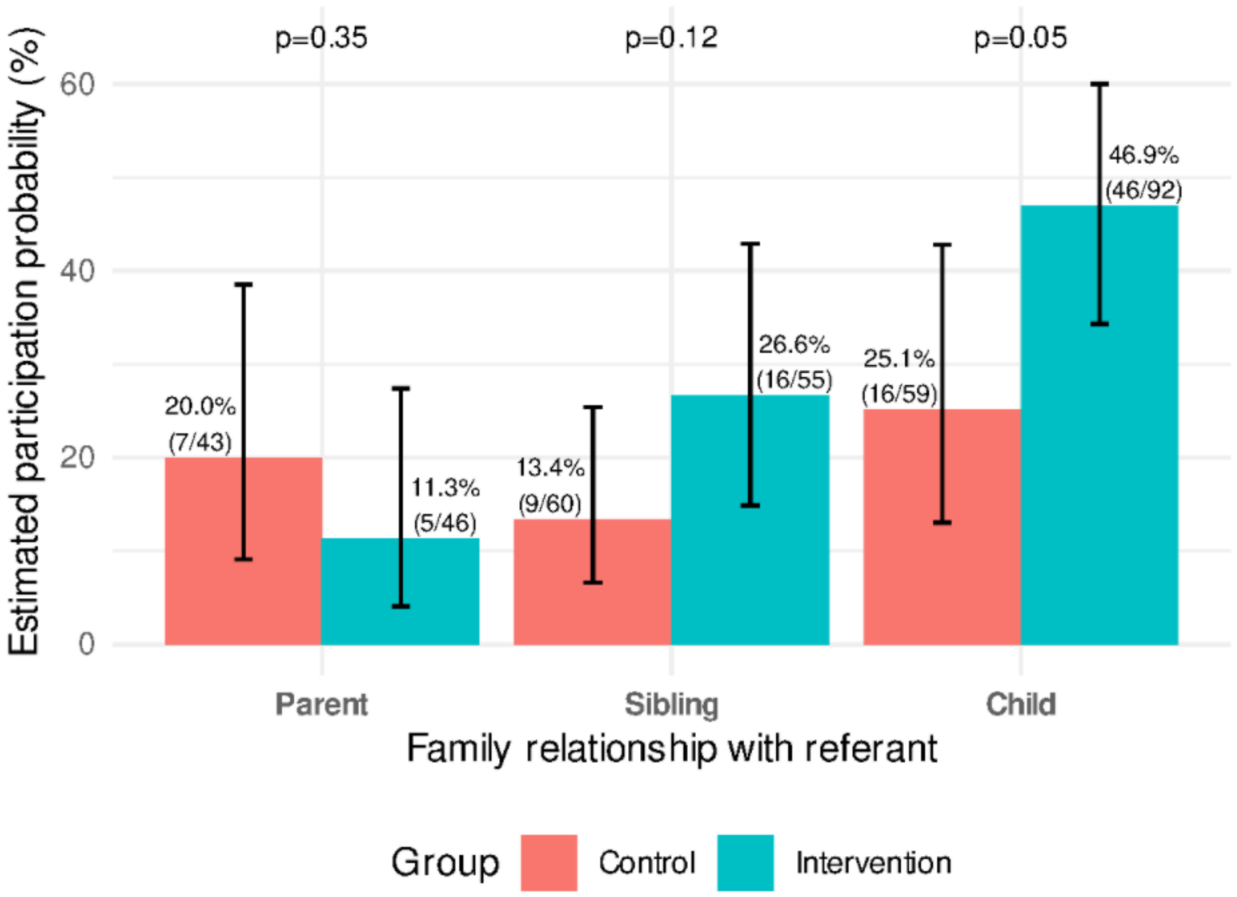

© 2026 Nanchen D et al. *JAMA Network Open*.

eFigure 6: 6-month participation probability of relatives by screening cycle and by study arm

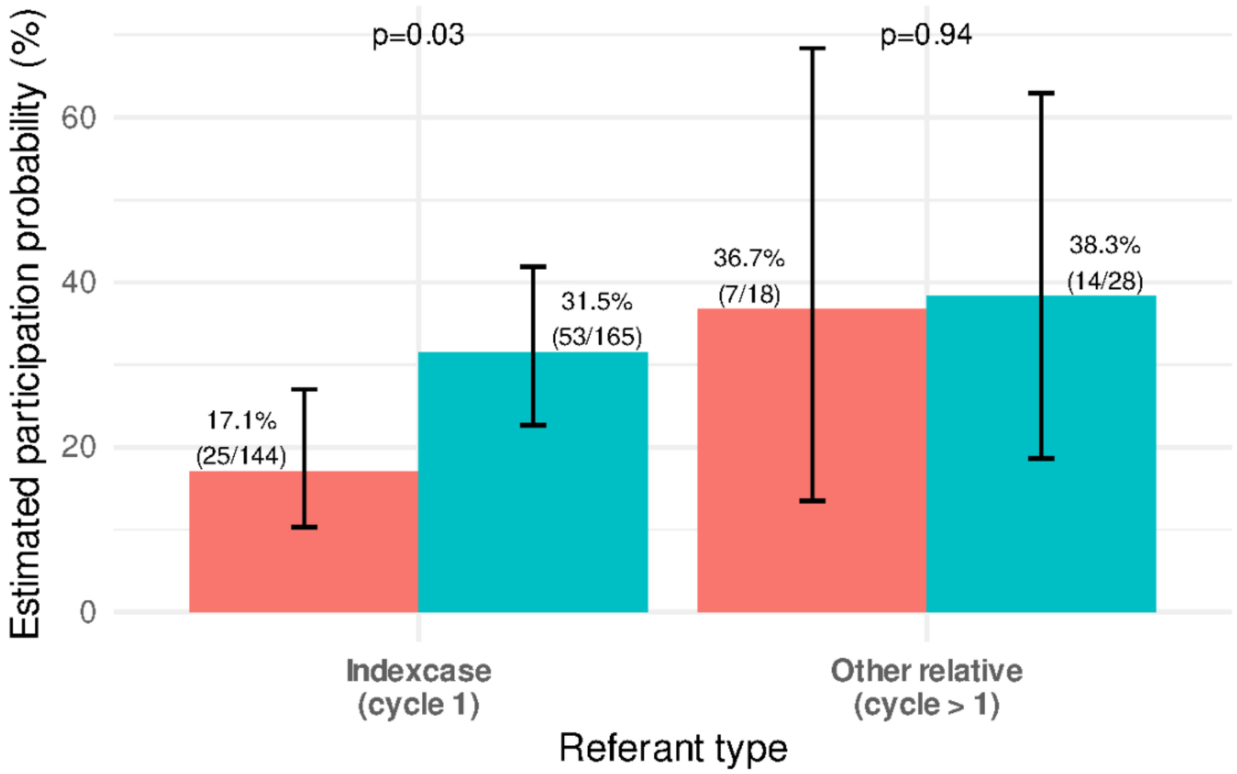

© 2026 Nanchen D et al. *JAMA Network Open*.
