# Supplementary material for: Genetic Cascade Screening for Familial Hypercholesterolemia: A Randomized Clinical Trial
Source: JAMA Netw Open. 2026 Apr 13;9(4):e266100. doi: 10.1001/jamanetworkopen.2026.6100 (PMC13077512; doi:10.1001/jamanetworkopen.2026.6100)
Supplement: Supplement 3. — Data Sharing Statement [file jamanetwopen-e266100-s003.pdf]

Data Sharing Statement

Nanchen. Genetic Cascade Screening for Familial Hypercholesterolemia. *JAMA Netw Open*. Published April 13, 2026. doi:10.1001/jamanetworkopen.2026.6100

Data
Additional Information: Trial Registration: NCT04419090, first submitted 2020-06-03
https://clinicaltrials.gov/study/NCT04419090
Data available: Yes
Data types: Deidentified participant data
How to access data: De-identified participant data (including the data dictionary), statistical code, and related materials are available via the Unisanté data repository upon reasonable request and subject to approval by the study scientific committee and relevant ethics committees.
When available: With publication

Supporting Documents
Document types: Other (please specify)
Additional Information: Codebook/data dictionnary, study-level metadata
How to access documents: The access request should be made through the Unisanté data repository : https://data.unisante.ch/home
When available: With publication

Additional Information
Who can access the data: researchers whose proposed use of the data has been approved
Types of analyses: for a specified purpose
Mechanisms of data availability: after approval of a proposal and with a signed data access agreement
